# Critical role of ROCK1 in AD pathogenesis via controlling lysosomal biogenesis and acidification

**DOI:** 10.1186/s40035-024-00442-9

**Published:** 2024-11-04

**Authors:** Chenghuan Song, Wanying Huang, Pingao Zhang, Jiyun Shi, Ting Yu, Jing Wang, Yongbo Hu, Lanxue Zhao, Rui Zhang, Gang Wang, Yongfang Zhang, Hongzhuan Chen, Hao Wang

**Affiliations:** 1 Department of Pharmacology and Chemical Biology, Shanghai Jiao Tong University School of Medicine, Shanghai, 200025 China; 2 Academy of Integrative Medicine, Shanghai University of Traditional Chinese Medicine, Shanghai, 201203 China; 3 Department of Neurology, Chang-Hai Hospital, The Second Military Medical University, Shanghai, 200433 China; 4 Department of Neurology and Neuroscience Institute, Ruijin Hospital Affiliated to Shanghai Jiao Tong University School of Medicine, Shanghai, 200025 China; 5 Shuguang Lab of Future Health, Shanghai Frontiers Science Center of TCM Chemical Biology, Shuguang Hospital, Shanghai University of Traditional Chinese Medicine, Shanghai, 201203 China

**Keywords:** ROCK1, Lysosomal biogenesis, Lysosomal acidification, TFEB, Alzheimer’s disease

## Abstract

**Background:**

Lysosomal homeostasis and functions are essential for the survival of neural cells. Impaired lysosomal biogenesis and acidification in Alzheimer’s disease (AD) pathogenesis leads to proteolytic dysfunction and neurodegeneration. However, the key regulatory factors and mechanisms of lysosomal homeostasis in AD remain poorly understood.

**Methods:**

ROCK1 expression and its co-localization with LAMP1 and SQSTM1/p62 were detected in post-mortem brains of healthy controls and AD patients. Lysosome-related fluorescence probe staining, transmission electron microscopy and immunoblotting were performed to evaluate the role of ROCK1 in lysosomal biogenesis and acidification in various neural cell types. The interaction between ROCK1 and TFEB was confirmed by surface plasmon resonance and in situ proximity ligation assay (PLA). Moreover, we performed AAV-mediated ROCK1 downregulation followed by immunofluorescence, enzyme-linked immunosorbent assay (ELISA) and behavioral tests to unveil the effects of the ROCK1–TFEB axis on lysosomes in APP/PS1 transgenic mice.

**Results:**

ROCK1 level was significantly increased in the brains of AD individuals, and was positively correlated with lysosomal markers and Aβ. Lysosomal proteolysis was largely impaired by the high abundance of ROCK1, while ROCK1 knockdown mitigated the lysosomal dysfunction in neurons and microglia. Moreover, we verified ROCK1 as a previously unknown upstream kinase of TFEB independent of m-TOR or GSK-3β. ROCK1 elevation resulted in abundant extracellular Aβ deposition which in turn bound to Aβ receptors and activated RhoA/ROCK1, thus forming a vicious circle of AD pathogenesis. Genetically downregulating ROCK1 lowered its interference with TFEB, promoted TFEB nuclear distribution, lysosomal biogenesis and lysosome-mediated Aβ clearance, and eventually prevented pathological traits and cognitive deficits in APP/PS1 mice.

**Conclusion:**

In summary, our results provide a mechanistic insight into the critical role of ROCK1 in lysosomal regulation and Aβ clearance in AD by acting as a novel upstream serine kinase of TFEB.

**Supplementary Information:**

The online version contains supplementary material available at 10.1186/s40035-024-00442-9.

## Introduction

In neural cells, autophagosomes fuse with lysosomes hosting a large number of hydrolases to form autolysosomes, which are mainly responsible for the autophagic degradation of protein aggregates and organelles, termed the autophagy-lysosomal pathway (ALP) [1–3]. Dysfunction of lysosomes, including reduced number and destructed acidic environment, leads to ALP disturbance and consequent proteotoxicity in age-related neurodegenerative diseases such as Alzheimer’s disease (AD) [4, 5]. Impaired lysosomal biogenesis and acidification, which has been observed in the brains of AD patients, can lead to insufficient clearance of Aβ peptides and tau protein, two major hallmarks of AD [6, 7]. Risk genes associated with late-onset AD such as *APOE*, *PSEN1*, *SORL1*, and *TREM2* are closely related to lysosomal pathways [8], also supporting the causal link between lysosomal disorders and AD. The genetic and clinical evidence underscores the significance of maintaining a steady state of lysosomal function in addressing this catastrophic disease.

Although promoting lysosomal homeostasis has been widely recognized as beneficial for the treatment of AD, knowledge remains limited concerning the pivotal genetic or epigenetic lysosomal regulators. ROCK1 is a serine/threonine kinase expressed in both neurons and glial cells in human brains. ROCK1 has well-known regulatory functions in actin cytoskeleton, cell proliferation and apoptosis [9–11]. Recently, we and others have reported new functions of ROCK1 in modulating Aβ and tau pathology, finding that reduction of ROCK1 diminishes both Aβ and tau levels in the brains of AD mice [12–14]. Since lysosomes are the main organelles for Aβ and tau degradation in neural cells, it would be interesting to explore whether ROCK1 manipulates ALP degradation of these toxic proteins by controlling lysosomal biogenesis and acidification.

Here we present data showing the potential effects of ROCK1 on lysosomal regulation and Aβ pathologies. We found that the superabundance of ROCK1 decreased lysosomal numbers and impaired lysosomal acidification, leading to cellular proteostasis disturbance, whereas inhibition of ROCK1 increased the lysosomal formation and maintained lysosomal acid environment. In addition, ROCK1 directly bound to and phosphorylated TFEB, but not TFE3 or ZKSCAN3, on Ser211 and Ser142 sites independent of m-TOR and GSK-3β. This reduced nuclear translocation of TFEB and its interaction with coordinated lysosomal expression and regulation (CLEAR) elements, thus affecting lysosomal formation, pH and enzyme content. Either mutation of TFEB at Ser211 and Ser142 which were elevated in the brains of AD patients, or knockdown of TFEB, was sufficient to abolish the effect of ROCK1 on lysosomes. Adeno-associated virus (AAV)-mediated downregulation of ROCK1 in the hippocampus elevated TFEB nuclear distribution, promoted lysosomal function and Aβ degradation, and consequently ameliorated the cognitive deficits in APP/PS1 mice.

## Methods

### Human brain samples for immunoblotting

**Fig. 1:**
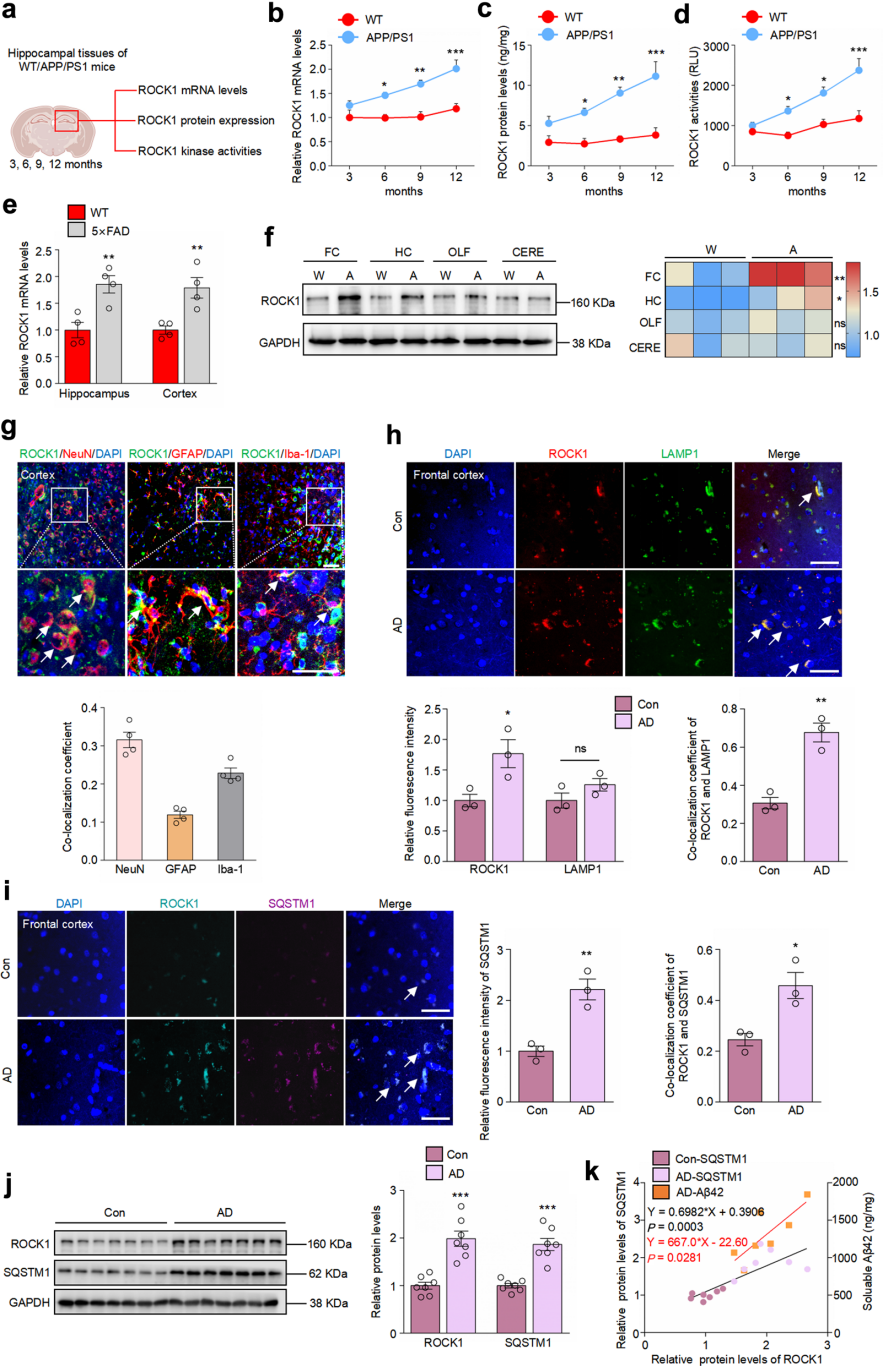
ROCK1 is upregulated in AD brains. **a** Schematic illustration of the experimental procedure created with BioRender.com. **b** qRT-PCR analysis of ROCK1 mRNA expression in the hippocampus of WT and APP/PS1 mice at different ages. *n* = 3. **c** ELISA analysis of the protein levels of ROCK1 in the hippocampus of WT and APP/PS1 mice at different ages. *n* = 3. **d** ROCK1 activity in the hippocampus of WT and APP/PS1 mice at different ages. **e** qRT-PCR analysis of mRNA expression of ROCK1 in the hippocampus and cortex of 9-month WT and 5 × FAD mice. *n* = 4. **f** ROCK1 protein levels in different brain regions of 9-month WT and APP/PS1 mice were detected by immunoblotting. *n* = 3. FC: frontal cortex, HC: hippocampus, OLF: olfactory bulb, CERE: cerebellum. Red color indicates higher expression; blue color indicates lower expression. **g** Co-localization of ROCK1 (green) with neurons (NeuN), astrocytes (GFAP), and microglia (Iba-1) in the cortex of 6-month-old WT mice. Nuclei were stained with DAPI (blue). White arrows indicate co-localization. *n* = 4. Scale bar, 50 μm. **h** Immunofluorescence staining of ROCK1 and LAMP1, and analysis of their co-localization in the frontal cortex of healthy controls and AD patients. *n* = 3. ns, no significance. Scale bars, 50 μm. **i** Immunofluorescence staining of SQSTM1 and ROCK1, and analysis of their co-localization in the frontal cortex of healthy controls and AD patients. *n* = 3. Scale bars, 50 μm. **j** The protein expression of ROCK1 and SQSTM1 in the temporal cortex of healthy controls and AD patients was analyzed by Immunoblotting. *n* = 7. **k** Scatter plot of soluble Aβ_42_ and relative SQSTM1 levels versus relative expression of ROCK1 in the human temporal cortex. Soluble Aβ_42_ levels were determined by ELISA in the same samples used in **j**. ROCK1 level was positively correlated with the levels of Aβ_42_ (orange line) and SQSTM1 (black line) determined by Spearman’s rank correlation test. *n* = 7. **b**–**d**, *P < 0.05, **P < 0.01, ***P < 0.001 versus WT group, two-way ANOVA followed by Bonferroni test. **e**, **f**, *P < 0.05, **P < 0.01 versus WT group, Unpaired two-tailed Student’s *t*-test. **h**–**j**, *P < 0.05, **P < 0.01, ***P < 0.001 versus Con group, Unpaired two-tailed Student’s* t*-test

Brain tissues used for immunoblotting in Figs. 1j and 4i were provided by the Netherlands Brain Bank (NBB), Netherlands Institute for Neuroscience, Amsterdam (www.brianbank.nl). Written informed consent for brain autopsy and the use of the material and clinical information for research purposes had been obtained from the donors by the NBB. Temporal cortical regions were used for immunoblotting. The healthy control donors (*n* = 7) did not have any mental or metabolic diseases, and the AD patient donors (*n* = 7) did not suffer from other neurological diseases according to neuropathological examination. Detailed information of the brain donors is described in Additional file 1: Table S1.

### Paraffin human brain sections used for immunofluorescence staining and proximity ligation assay (PLA)

Paraffin sections of human brain used for immunofluorescence staining and PLA were provided by the National Human Brain Bank for Development and Function, Chinese Academy of Medical Sciences, Beijing, China (http://anatomy.sbm.pumc.edu.cn/). Written informed consent for brain autopsy and the use of the material and clinical information for research purposes had been obtained by the National Human Brain Bank for Development and Function. Frontal cortical samples of 3 healthy controls and 3 AD cases were used. Detailed information of the brain donors is listed in Additional file 2: Table S2.

### Animals

Transgenic mice overexpressing hAPP695swe (APP) and presenilin-1M146V (PS1) mutations (APP/PS1) and wild-type (WT) C57BL/6J mice were purchased from the Model Animal Research Center (MARC) of Nanjing University (Nanjing, China). 5 × FAD mice were obtained from the Jackson Laboratory (Bar Harbor, ME). The mice were kept at a constant temperature (24 ± 2 °C) with free access to tap water and food. The mice used in our experiments were all males and the numbers of animals used in each experiment are shown in the corresponding figure legends. All mice were randomly allocated to different groups and investigators were blinded to the group allocation. No mice were excluded in the experiments. All animal experimental procedures were approved by the Ethics Committee of Shanghai Jiao Tong University School of Medicine.

### Stereotactic injection of AAV

To knock down ROCK1 in the brains of both WT and APP/PS1 mice, 7.5-month-old mice were anesthetized by inhalation of 2.5% isoflurane and positioned in a stereotaxic apparatus. The AAV carrying either control shRNA with GFP (AAV-sh-Con) (4 µl, 5.8 × 10^12^ viral genomes/µl, Sangon Biotech, Shanghai, China) or the ROCK1 shRNA with GFP (AAV-sh-ROCK1) (4 µl, 4.4 × 10^12^ viral genomes/µl, Sangon Biotech) was microinjected into the hippocampus of mice using the following microinjection coordinates: anteroposterior − 2.06 mm, lateral ± 1.5 mm, and ventral + 2.0 mm (stereotaxic coordinates from bregma). Similarly, to overexpress ROCK1, 9-month-old WT mice were microinjected with AAV preparations carrying either Control-GFP (AAV9-Con) or ROCK1 plasmids (AAV-ROCK1) (3 µl, 1.0 × 10^13^ viral genomes/µl, SunBio Biomedical Technology Co., Ltd, Shanghai, China) into the hippocampus at the same coordinates after anesthesia. AAV9 viruses were designed and constructed under the CAG promoter.

### Cell line cultures, transfection and reagents

HEK-293T, PC12 and BV-2 cells were purchased from American Type Culture Collection (ATCC, Mannesas, VA) and maintained in Dulbecco’s modified Eagle’s medium (DMEM, Thermo Fisher Scientific, Waltham, MA) with 10% heat-inactivated fetal bovine serum (FBS; Gibco, Logan, UT) and 1% penicillin–streptomycin (Thermo Fisher Scientific) at 37 °C in a 5% CO_2_ incubator. ROCK1 WT and truncated plasmids, TFEB-WT, TFEB-MS211 and TFEB-MS142 plasmids, as well as siRNAs targeting ROCK1, TFEB, TFE3 and ZKSCAN3 were transfected into cells by using Lipofectamine 3000 (Invitrogen, Carlsbad, CA) according to the manufacturer’s instructions. m-TOR inhibitor Torin1 was purchased from Tocris Bioscience (4247, Bristol, England). GSK3β inhibitor SB415286 was purchased from Abcam (ab120962, Cambridge, England). Sequences of siRNAs used in this study are shown in Additional file 1: Table S3.

### Primary mouse neuronal and microglial cultures and lentivirus infection

Mouse neurons were obtained from the cortices of postnatal day 0 (P0)–P1 C57BL/6 mice purchased from Nanjing Model Animal Resource Center (Nanjing, China). Briefly, cortical tissues were dissected in ice-cold PBS with 15% FBS, NaHCO_3_ (4 mM), and HEPES (1 mM). Neurons were then pelleted, resuspended and plated on a coverslip coated with poly-Ornithine (0.1 mg/ml, Sigma-Aldrich, St. Louis, MO) and laminin, then cultured with neurobasal supplemented with 10% FBS (Gibco), B27 (1 ×), glutamate, and Pen/Strep (0.5 mg/ml) at 37 °C under a humidified 5% CO_2_ atmosphere. For lentivirus transduction, neurons were plated onto a 6-well plate coated with poly-*L*-lysine (100 µg/ml, Sigma-Aldrich) at a density of 1 × 10^6^ cells/well. The neurons were cultured for 14–16 days and then transduced with lentivirus carrying either the control shRNA (LV-sh-Con) or the ROCK1 shRNA (LV-sh-ROCK1) (1.0 × 10^9^ viral genomes/µl, Genomeditech Co., LTD, Shanghai, China). For microglia, cells were pelleted, and then resuspended in DMEM supplemented with 10% FBS and 1% penicillin–streptomycin mix. The microglia were isolated by shaking and collected from the medium by centrifugation at 1500× *g* for 5 min. The procedures of lentivirus transduction were the same as in neurons.

### Cytoplasmic and nuclear protein extraction

Cytoplasmic and nuclear protein extraction was carried out using a commercial kit (Beyotime, Shanghai, China) following the manufacturer's instructions. Briefly, hippocampal tissue samples were cut into small pieces and placed into a tube, followed by homogenizing in cytoplasmic protein extraction reagent. Then they were placed on ice for 15 min and centrifuged at 1500 × *g*, 4 °C for 5 min, and supernatant was obtained as cytoplasmic protein. The nuclear protein extraction reagent was added into the precipitation, and the supernatant was obtained as nuclear protein after 16,000 × *g* centrifugation for 10 min at 4 °C.

### Immunoblotting

Mouse brain tissues and cell samples were lysed in RIPA buffer (Beyotime), and immunoblotting was carried out according to our previous procedures [15]. The antibodies used for immunoblotting are as follows: ROCK1 (Abcam, ab134181), SQSTM1 (Abcam, ab56416), myc (Abcam, ab18185), lysosome-associated membrane glycoprotein (LAMP) 1 (Abcam, ab24170), LAMP2 (Abcam, ab25631), Cathepsin B (CtsB) (Cell Signaling Technology, Danvers, MA, 31718T), CtsD (Cell Signaling Technology, 2284S), ATP6V1A (Abcam, ab199326), Rab5 (Abcam, ab18211), Rab7 (Abcam, ab126712), IGF2R (Abcam, ab124767), mannose-6-phosphate receptor (M6PR) (Abcam, ab134153), TFEB (Abcam, ab2636), p-TFEB Ser211 (Affinity, Melbourne Australia, AF3708), p-TFEB Ser142 (Millipore, Billerica, MA, ABE1971-I), p-TFEB Ser122 (Cell Signaling Technology, 86843), Histone H3 (Abcam, ab1791), and GAPDH (Abcam, ab181602).

### Real‐time RT-PCR

Total RNA was extracted from mouse brain tissues and cells using a SteadyPure Universal RNA Extraction Kit (Accurate Biotechnology Co., Ltd, Changsha, China). The extracted RNA samples were reverse-transcribed into cDNA using a PrimeScript 1st Strand cDNA Synthesis Kit (TaKaRa Biotechnology, Kusatsu, Japan) according to the manufacturer’s instructions. RT-PCR was carried out with TB Green Premix Ex Taq (TaKaRa Biotechnology) and run by the LightCycler480 System (Roche, Switzerland). The primer sequences are shown in Additional file 1: Table S4.

### Immunofluorescence

Immunofluorescence was performed according to our previous procedures [16]. Briefly, mouse brain samples were fixed in 4% paraformaldehyde (PFA) and dehydrated in 20% and 30% sucrose in PBS. After that, the brain tissues were sectioned on a cryostat at 20 μm and stored in PBS-glycerin solution. Cells were washed with PBS three times for 5 min each and fixed in 4% PFA for 15 min at room temperature. The sections and cells were blocked with 5% fetal bovine serum in PBS at room temperature for 1 h, then incubated with the primary antibody at 4 °C overnight and the corresponding secondary antibody (1:1000) for 1 h at room temperature next day. For paraffin sections of human brain, the sections were heated at 60 °C for 30 min, and successively placed into xylene I for 10 min, xylene II for 10 min, anhydrous ethanol for 10 min, 85% alcohol for 10 min, and 70% alcohol for 10 min. Then the paraffin sections were subjected to antigen retrieval. After that, the sections of human brain were blocked and incubated with the primary antibody as mouse brain sections. The antibodies used for immunofluorescence in this study were ROCK1 (Abcam, ab134181), LAMP1 (Abcam, ab24170), SQSTM1 (Abcam, ab56416), Rab5 (Abcam, ab18211), Rab7 (Abcam, ab126712), TFEB (Abcam, ab2636), IGF2R (Abcam, ab124767), M6PR (Abcam, ab134153), ATP6V0D1 (Abcam, ab56441), ATP6V1A (Abcam, ab19926), Aβ (Cell Signaling Technology, 2450T), GFAP (Abcam, ab7260), and Iba-1 (Abcam, ab178847).

### LysoTracker red staining

The LysoTracker Red DND-99 probe (50 nM, Invitrogen) was used to identify acidic lysosomes. Cells were incubated with the medium containing LysoTracker Red DND-99 probe for 30 min at 37 °C, then the medium was replaced by fresh normal culture medium, and fluorescent images were captured using a Leica SP8 confocal microscope (Leica, Wetzlar, Germany).

### Cathepsin B (CtsB) activity assay

CtsB activity was determined using the Magic Red B probe (Invitrogen) according to the manufacturer’s protocol. After the cells had grown to the appropriate confluence, 100 nM Magic Red B probe was added to the medium and incubated for 30 min at 37 °C. Then the supernatant was replaced with the fresh medium and the resulting fluorescence was observed using a Leica SP8 confocal microscope.

### DQ-BSA staining

For proteolysis assays with lysosome-loaded DQ-BSA, cells were loaded with 0.2 mg/ml DQ-BSA probe (Invitrogen) for 2 h, and then washed with PBS twice. Cells were chased in fresh normal medium for an additional 2 h to allow lysosomal loading of DQ-BSA. Fluorescent images were then obtained by using a Leica SP8 confocal microscope.

### Lysosomal pH measurement

Lysosomal pH was determined with LysoSensor Yellow/Blue DND-160 dextran (Invitrogen). When cells reached the desired confluence, the medium was replaced with pre-warmed (37 °C) LysoSensor Yellow/Blue DND-160 dextran-containing medium, and then incubated for 6 h in the incubator. The loading solution was replaced with fresh medium and fluorescence emission was recorded at 440 nm/540 nm using a Synergy H1 plate reader (BioTek, Winooski, VT). To generate the lysosomal pH calibration curve, cells were incubated with pH calibration buffers (ranging from pH 4.5–7.5) supplemented with nigericin and monensin at 37 °C for 30 min to establish a calibration curve. The pH values were calculated based on the corresponding 440 nm/540 nm ratios.

### ELISA

Hippocampal tissues of APP/PS1 mice microinjected with AAV-sh-Con/AAV-sh-ROCK1 or human brain tissues were homogenized in PBS to obtain the soluble proteins and in guanidine to gain the insoluble component. Aβ_40_ was detected with a commercially available ELISA kit (Raybiotech, Atlanta, GA), and Aβ_42_ was determined by another kit (Biolegend, San Diego, CA) according to each manufacturer's instructions. Plates were read at 450 nm on a Synergy MX Plate Reader (BioTek).

### Flow cytometry

Mouse primary microglia transduced with LV-sh-Con/LV-sh-ROCK1 were incubated with FITC-Aβ for 6 h. After three PBS washes, cells were suspended in PBS and transferred into tubes for quantification of FITC-Aβ-positive cells by flow cytometry in Attune NxT (Invitrogen).

### Transmission electron microscopy

Approximately 1-mm^3^ mouse hippocampal samples were collected and fixed in 2.5% glutaraldehyde (Sigma-Aldrich) in PBS. After that, samples were osmicated in 2% osmium tetroxide (Sigma-Aldrich) in PBS and dehydrated in a graded series of ethanol. The samples were then embedded with epoxy resin and cut into sections of 70 nm thickness with an ultramicrotome (Leica, Wetzlar, Germany). The sections were then stained with 2% uranyl acetate and lead citrate. Lysosomes were observed with a transmission electron microscope (HITACHI, H-7650, Japan) operated at 80 kV. Similarly, cells were fixed, osmicated, dehydrated, embedded in the solutions mentioned above, and then examined under the transmission electron microscope.

### Surface plasmon resonance (SPR)

TFEB was immobilized on the surface of the CM5 chip using the amine-coupling approach. The sensor surface was activated with a mixture of 50 mM *N*-hydroxysuccinimide and 200 mM 1-ethyl-3-(3-dimethylaminopropyl) carbodiimide. ROCK1 was then injected at serial concentrations from 312.5 to 5000 nM for 90 s. The gained data were fitted and analyzed using the Biacore T200 software (GE Healthcare, Chicago, IL).

### Proximity ligation assay (PLA)

Paraffin sections of human brain were dewaxed and antigen-fixed. Then PLA was performed using the Duolink in situ PLA Kit (Red) (Sigma-Aldrich) according to the manufacturer's instructions. Briefly, the sections were blocked for 1 h at 37 °C and incubated with primary antibodies diluted in the Duolink® antibody diluent at 37 °C overnight. The next day, the sections were incubated with the Duolink® PLUS and MINUS PLA probe for 1 h at 37 °C, followed by ligation and amplification. HEK-293T cells were washed with PBS for three times, blocked and incubated with primary antibodies. The following steps were the same as brain sections mentioned above. Images were taken using a Leica TCS SP8 laser confocal microscope. ROCK1 (Abcam, ab45171) and TFEB (Abcam, ab2636) antibodies were used.

### Co-immunoprecipitation

HEK-293T cells were lysed in Pierce™ IP lysis buffer (Thermo Fisher Scientific) with a protease inhibitor cocktail (APExBIO Technology, Houston, TX). Anti-ROCK1 antibody was added to the obtained samples and incubated at 4 °C overnight. The lysates were incubated with protein A/G agarose resin (Santa Cruz Biotechnology, Dallas, TX) on a rotator for 2 h the next day. Proteins were eluted from the resin with 2 × SDS sample buffer and used for immunoblotting analysis.

### Y-maze spontaneous alternation test

Y-maze spontaneous alternation test was carried out 4 weeks after microinjection of AAV-sh-Con or AAV-sh-ROCK1 in the APP/PS1 and WT mice. On the day of the test, all mice were transferred to the room 1 h before testing to adapt to the environment. The trial was conducted in an opaque Perspex Y-maze with the arms spaced at an angle of 120°. Each mouse was placed at the end of arm A and explored the maze freely for 10 min. After each exploration, the Y-maze was cleaned with 70% ethanol. Spontaneous alternation was defined as a successive entry into three different arms.

### Novel object recognition test

Before experiment, mice were placed into an open field (60-cm long and 60-cm wide with walls of 60-cm high) for 10 min. Then two identical objects were positioned in the open field and each mouse was allowed to explore the two objects freely for 10 min. After 24 h, one of the objects was replaced by a totally different new object. Each mouse was allowed 10 min to explore the objects. After each exploration, the chamber was wiped with 70% ethanol. The preference of each mouse for the two objects was recorded by a video camera.

### Barnes maze

The Barnes Maze consisted of a rotatable circular platform (1.22 m in diameter and 1 m from the floor) with 20 holes in the periphery. A removable box was placed underneath one of the holes for escape, and visual cues were placed on the walls of the room. Mice were transported from their cages to the center of the platform via a closed starting chamber where they stayed for 10 s prior to exploring the maze for 3 min. Mice failing to enter the escape box within 3 min were guided to the escape box, and the latency was counted as 180 s. Mice were allowed to stay in the escape box for 10 s before the next trial. Each mouse underwent two trials per day for 7 consecutive days. The platform and the escape box were wiped with 70% ethanol after each trial to eliminate the olfactory cues of the target hole. All trials were recorded by a video camera and analyzed with the tracking software.

### Statistical analysis

Statistical analysis was performed using GraphPad Prism 7 and all data are presented as mean ± standard error of mean (SEM). Two-sided unpaired Student’s* t*-test was used for two‐group comparisons. Comparisons among groups were analyzed by ANOVA followed by Bonferroni’s *post-hoc* multiple comparison tests. Pearson correlation analysis was performed to calculate correlations. Sample sizes were determined according to our previous studies and other similar publications in reasonable sample sizes for different experiments. *P* < 0.05 was deemed as statistically significant.

## Results

### ROCK1 is upregulated in human and mouse AD brains

To determine the changes of ROCK1 expression and activity throughout the pathological process of AD, we analyzed the mRNA expression, protein level and kinase activity of ROCK1 in the hippocampus of WT and APP/PS1 mice at 3, 6, 9, and 12 months of age (Fig. 1a). The results showed that ROCK1 level and activity were only slightly increased with age in the hippocampus of WT mice, while in APP/PS1 mice they started to be upregulated at 6 months when Aβ burden appeared and showed an age-dependent elevation (Fig. 1b–d). ROCK1 mRNA expression was also significantly elevated in the hippocampus and cortex of 9-month-old 5 × FAD mice compared to WT mice (Fig. 1e). We also analyzed differentially expressed genes in the brains of AD patients compared to healthy controls in some databases. ROCK1 expression in both frontal and temporal cortex of AD patients was much higher than that of controls (Additional file 2: Fig. S1a, b). No significant difference in ROCK1 expression was detected between males and females in either control or AD group (Fig. S1c–f). We further analzyed ROCK1 levels in different brain regions and cell types. There was no significant difference in the expression of ROCK1 among four brain regions of WT mice, while ROCK1 was particularly increased in the cortex and hippocampus of APP/PS1 mice (Fig. 1f). Moreover, ROCK1 was pervasively present in various cell types, but was more highly expressed in neurons and microglia (Fig. 1g).

To determine whether ROCK1 is correlated with lysosomes, protein–protein interaction (PPI) analysis was carried out. ROCK1 has a correlation with many known lysosomal proteins such as LAMP1 and LAMP2 [17, 18] (Fig. S1g). We further validated these results in the cortex of individuals with or without AD. Consistent with what we observed in mouse models, ROCK1 was upregulated in the frontal (Fig. 1h) and temporal cortex (Fig. 1j) of AD patients compared to controls. Besides, co-localization of ROCK1 with LAMP1 or SQSTM1/p62 (an autophagic substrate) [19] was found in control brains, and significantly increased in AD individuals (Fig. 1h, i). The expression of ROCK1 was positively correlated with that of SQSTM1 (Fig. 1j, k), whose abnormal accumulation in AD represents an impeded autophagy flux and degradation of toxic proteins [20, 21]. We therefore measured Aβ_42_ in the temporal cortex of AD patients and found a positive correlation between ROCK1 and Aβ_42_ levels (Fig. 1k). Taken together, the results above indicated that the increase of ROCK1, observed in both AD patients and AD mice, is linked with impaired autophagy and subsequent Aβ accumulation.

### ROCK1 impairs lysosomal biogenesis and acidification

**Fig. 2:**
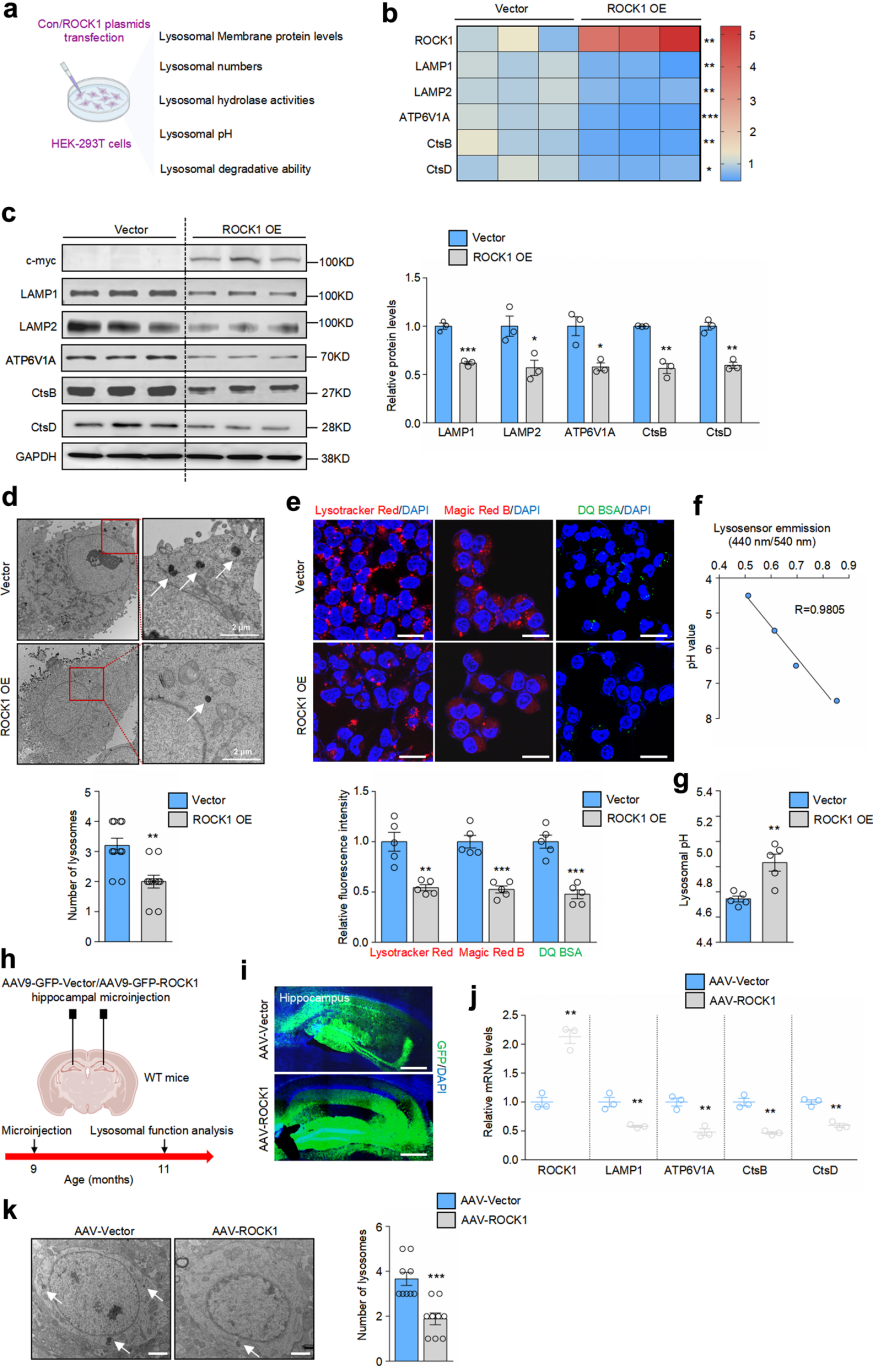
ROCK1 decreases lysosomal biogenesis and impairs lysosomal acid environment. **a** Schematic illustration of the experimental procedure. Image created with BioRender.com. **b** Heatmap of mRNA expression of *ROCK1* and lysosome-related markers detected with qRT-PCR in HEK-293T cells transfected with control or ROCK1-overexpression plasmid for 48 h. *n* = 3. Red color, higher expression; blue color, lower expression. **c** Immunoblotting of lysosome-related markers in HEK-293T cells and quantitation of their protein levels. *n* = 3. **d** Transmission electron microscopy of lysosomes in HEK-293T cells. The number of lysosomes was analyzed. *n* = 10 cells per group. Scale bar, 2 μm. White arrows indicate lysosomes. **e** Lysotracker Red, Magic Red B and DQ BSA staining in HEK-293T cells. Fluorescence intensities were analyzed. *n* = 5. Scale bars, 25 μm. **f** Standard curve of LysoSensor to determine the lysosomal pH values of HEK-293T cells. **g** Lysosomal pH of HEK-293T cells was determined by LysoSensor. *n* = 5. **h** Schematic illustration of the experimental procedure. Image created with BioRender.com. **i** AAV-Vector and AAV-ROCK1 were successfully microinjected into the hippocampus of WT mice, respectively. GFP (green) was used to visualize viral diffusion. Scale bar, 200 μm. **j** qRT-PCR analysis of expression levels of indicated mRNAs. *n* = 3. **k** Transmission electron microscopy of lysosomes in the hippocampus. The number of lysosomes was analyzed. *n* = 9 slices from 3 mice. Scale bars, 1 μm. *P < 0.05, **P < 0.01, ***P < 0.001 versus vector (**b**-**e**, **g**) or AAV-Vector group (**j**, **k**), Student’s *t*-test

To explore whether increased ROCK1 directly influences lysosomal function, HEK-293T cells were transfected with vector or c-myc-labeled ROCK1 overexpression plasmids, and the effects of ROCK1 on lysosomal biogenesis, hydrolase activity, pH value and degradative ability were evaluated (Fig. 2a). ROCK1 overexpression effectively decreased the mRNA expression and protein levels of both LAMP1 and LAMP2 (Fig. 2b, c). Transmission electron microscopy revealed that ROCK1 overexpression reduced the number of lysosomes, and decreased lysosome volume and contents compared with the control group (Fig. 2d).

Lysosomes have a highly acidic environment inside (pH between 4.5 and 5.0), which is essential for the normal functions of various acid hydrolases [22]. Besides, lysosomes control the internal pH through proton-pumping V-type ATPase (Vacuolar-type proton adenosine triphosphatase). Our data showed that ROCK1 overexpression decreased the expression of V-type proton ATPase catalytic subunit A (ATP6V1A) (Fig. 2b, c), suggesting that ROCK1 might impair the lysosomal acidic environment. To further test this possibility, Lysotracker Red and LysoSensor Yellow/Blue dextran staining was performed. Decreased acidic lysosomes and elevated pH values inside lysosomes were detected in ROCK1-overexpressing cells (Fig. 2e–g).

Considering that ROCK1 overexpression impaired lysosomal acidification, it was conceivable that lysosomal function would be undermined in ROCK1-overexpressing cells. Transfection of exogenous ROCK1 downregulated the levels of CtsB and CtsD that are considered indispensable for the degradation and turnover of intracellular proteins [23] (Fig. 2b, c). Consistently, the activity of CtsB was decreased by ROCK1 overexpression (Fig. 2e). Finally, we tested the effect of ROCK1 on lysosomal proteolytic ability by using DQ BSA, a probe that fluorescences upon degradation [24]. ROCK1 overexpression in HEK-293T cells obviously decreased the fluorescence intensity (Fig. 2e), indicating that ROCK1 reduces the ability of lysosomes to degrade engulfed foreign materials.

To consolidate the detrimental role of ROCK1 in lysosomes in vivo, we implemented AAV9-mediated ROCK1 overexpression in the hippocampus of WT mice (Fig. 2h). Two months after the microinjection, green fluorescent protein (GFP) signal was significantly dispersed in the hippocampus (Fig. 2i), and the mRNA level of ROCK1 was largely upregulated (Fig. 2j). Similar to the results in HEK-293T cells, ROCK1 overexpression caused strong reductions in the mRNA expression of LAMP1, ATP6V1A, CtsB and CtsD (Fig. 2j). Decreased lysosomal numbers were also observed in the ROCK1-overexpressing neurons (Fig. 2k). Taken together, these results indicated that ROCK1 overexpression undermines lysosomal biogenesis, acidification as well as storage and hydrolysis functions.

### Knockdown of ROCK1 promotes lysosomal biogenesis and maintains lysosomal acid environment

**Fig. 3:**
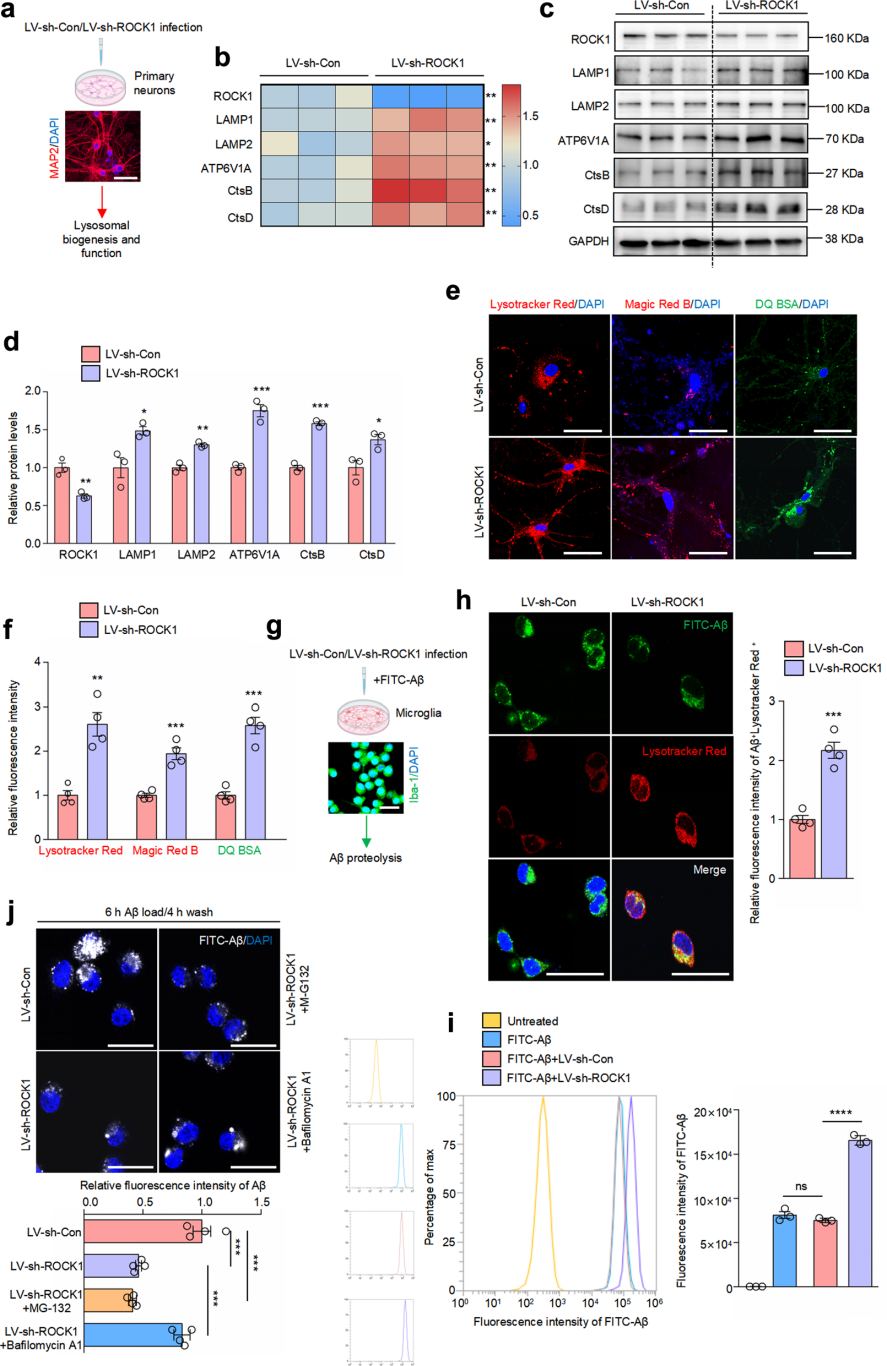
ROCK1 downregulation increases lysosomal numbers and maintains lysosomal acid environment. **a** Schematic illustration of the experimental procedure. Image created with BioRender.com. Primary mouse neurons were cultured and immunostained with anti-MAP2 antibody. Scale bar, 50 μm. **b** Primary mouse neurons were infected with lentiviral preparations expressing either sh-Con or sh-ROCK1 for 48 h. Heatmap of mRNA expression of indicated genes analyzed by qRT-PCR. *n* = 3. Red color, higher expression; blue color, lower expression. **c**, **d** Immunoblotting of indicated proteins in neurons infected with LV-sh-Con/LV-sh-ROCK1. *n* = 3. **e**, **f** Lysotracker Red, Magic Red B and DQ BSA staining in primary mouse neurons infected with LV-sh-Con/LV-sh-ROCK1. Relative fluorescence intensity was analyzed. *n* = 4. Scale bar, 50 μm. **g** Schematic illustration of the experimental procedure. Image created with BioRender.com. Primary mouse microglia were cultured and immunostained with anti-Iba-1 antibody. Scale bar: 50 μm. **h** Mouse primary microglia were infected with LV-sh-Con/LV-sh-ROCK1 for 48 h followed by incubation with FITC-labeled Aβ for 6 h, and further incubated with Lysotracker Red. *n* = 4. Scale bars, 50 μm. **i** Mouse primary microglia were infected with LV-sh-Con/LV-sh-ROCK1 for 48 h followed by incubation with FITC-labeled Aβ for 6 h. The population of FITC-Aβ-positive cells was measured by flow cytometery. *n* = 3. ns, no significance. **j** Mouse primary microglia infected with LV-sh-Con/LV-sh-ROCK1 were treated with MG-132 or Bafilomycin A1 for 1 h, followed by incubation with FITC-labeled Aβ for 6 h, washed for 4 h, and observed under a microscope. *n* = 4. Scale bars, 50 μm. **b**–**f**, *P < 0.05, **P < 0.01, ***P < 0.001 versus LV-sh-Con group. **i**, **j,** ***P < 0.001, ****P < 0.0001 versus indicated group. Student’s *t*-test

After revealing that lysosomal homeostasis is impaired upon ROCK1 increase, we further explored whether ROCK1 knockdown improves lysosomal biogenesis and function in neurons and microglia since ROCK1 is mainly expressed in these two cell types. Primary mouse cortical neurons were cultivated and infected with lentivirus carrying either the control shRNA (LV-sh-Con) or the ROCK1 shRNA (LV-sh-ROCK1) (Fig. 3a). Knockdown of ROCK1 significantly increased mRNA expression and protein levels of lysosomal membrane markers, V-ATPase component and hydrolases (Fig. 3b–d). ROCK1 downregulation also led to significant increases in the lysosomal acidotrophic fluorophores Lysotracker Red, the fluorescence of Magic Red B, and the degradation of DQ BSA probe (Fig. 3e, f).

Microglia can phagocytose extracellular Aβ and degrade them through acidic proteolytic enzymes in lysosomes [25]. After confirming that lentivirus-mediated ROCK1 knockdown also promoted lysosomal function in mouse primary microglia (Fig. S2a, b), we incubated microglia with FITC-labeled Aβ_42_ oligomers to detect Aβ degradation (Fig. 3g). ROCK1 downregulation obviously promoted the co-localization of FITC-Aβ_42_ and lysosomes (Fig. 3h). Flow cytometry experiment also indicated that ROCK1 downregulation promoted Aβ uptake by microglia (Fig. 3i). In Aβ degradation experiments, a larger portion of the fluorescent Aβ_42_ oligomers was degraded in the ROCK1-knockdown cells after a 4-h chase period, which could be reversed by Bafilomycin A1, a lysosome inhibitor, but not proteasome inhibitor MG-132 (Fig. 3j). This observation suggested that inhibition of ROCK1 enhances the degradative ability of Aβ through lysosomes.

We further verified ROCK1 functions in lysosomes in neuronal PC12 and microglial BV-2 cell lines. The knockdown efficiency of siRNAs targeting ROCK1 was validated (Fig. S3a–d). ROCK1 downregulation generally induced lysosomal biogenesis (Fig. S3e) and acidification (Fig. S3f) in both cell lines. It has been reported that the V-ATPase V1 domain moves from the cytoplasm to the lysosomal membrane-anchored V0 domain to assemble and activate the V-ATPase, resulting in the acidification of lysosomal lumen [26]. In addition to controlling the expression of ATP6V1A, ROCK1 also regulated V-ATPase assembly (Fig. S3g, h). Taken together, these results indicated that ROCK1 downregulation promotes lysosomal biogenesis and function, and is beneficial to the maintenance of the acidic environment of lysosomes in various types of cells, indicating that the underlying mechanism is conserved.

### ROCK1 regulation of lysosomal biogenesis is not mainly dependent on the endosome-lysosome pathway

Lysosome biogenesis is primarily regulated by the orchestra of transcription factor-controlled transcription of lysosomal genes (mainly manipulated by TFEB, TFE3 and ZKSCAN3) and endocytic transport of newly synthesized lysosomal membrane proteins (Fig. S4a) [27]. To uncover the exact roles of ROCK1 in the regulation of lysosomes, we first determined whether ROCK1 influences the synthesis and delivery of lysosomal proteins via the endosome-lysosome pathway. Double immunostaining of ROCK1 and organelle markers showed that ROCK1 was mainly colocalized with lysosomal marker LAMP1, but not with early endosomal marker Rab5 or late endosomal marker Rab7 (Fig. S4b). Newly synthesized lysosomal membrane proteins can be directly transported from the trans-Golgi network to endosomes in a M6PR-dependent manner, or indirectly delivered to the plasma membrane and internalized to the early endosomes [28, 29]. HEK-293T cells were treated with clathrin-dependent endocytosis inhibitor dynasore (Dyn) or macropinocytosis inhibitor 5-(*N*-ethyl-*N*-isopropyl) amiloride (EIPA) [30, 31], followed by ROCK1 knockdown. Neither Dyn nor EIPA treatment eliminated the effect of ROCK1 on LAMP1 expression (Fig. S4c), suggesting that endocytosis is not the primary course contributing to ROCK1-mediated lysosomal biogenesis.

We next examined whether ROCK1 directly binds M6PRs to deliver lysosomal proteins to endosomes [32]. The protein fluorescence signal of ROCK1 showed no obvious co-localization with cation-independent M6PR (also known as IGF2R) and cation-dependent M6PR (CD-M6PR) (Fig. S4d). Co-immunoprecipitation assays also confirmed that ROCK1 did not directly bind with IGF2R and CD-M6PR (Fig. S4e, f). Taken together, these results suggested that the influence of ROCK1 on lysosomal biogenesis is not primarily through the endosome-lysosome pathway.

### ROCK1 directly binds and phosphorylates TFEB, thus inhibiting its nuclear translocation

**Fig. 4:**
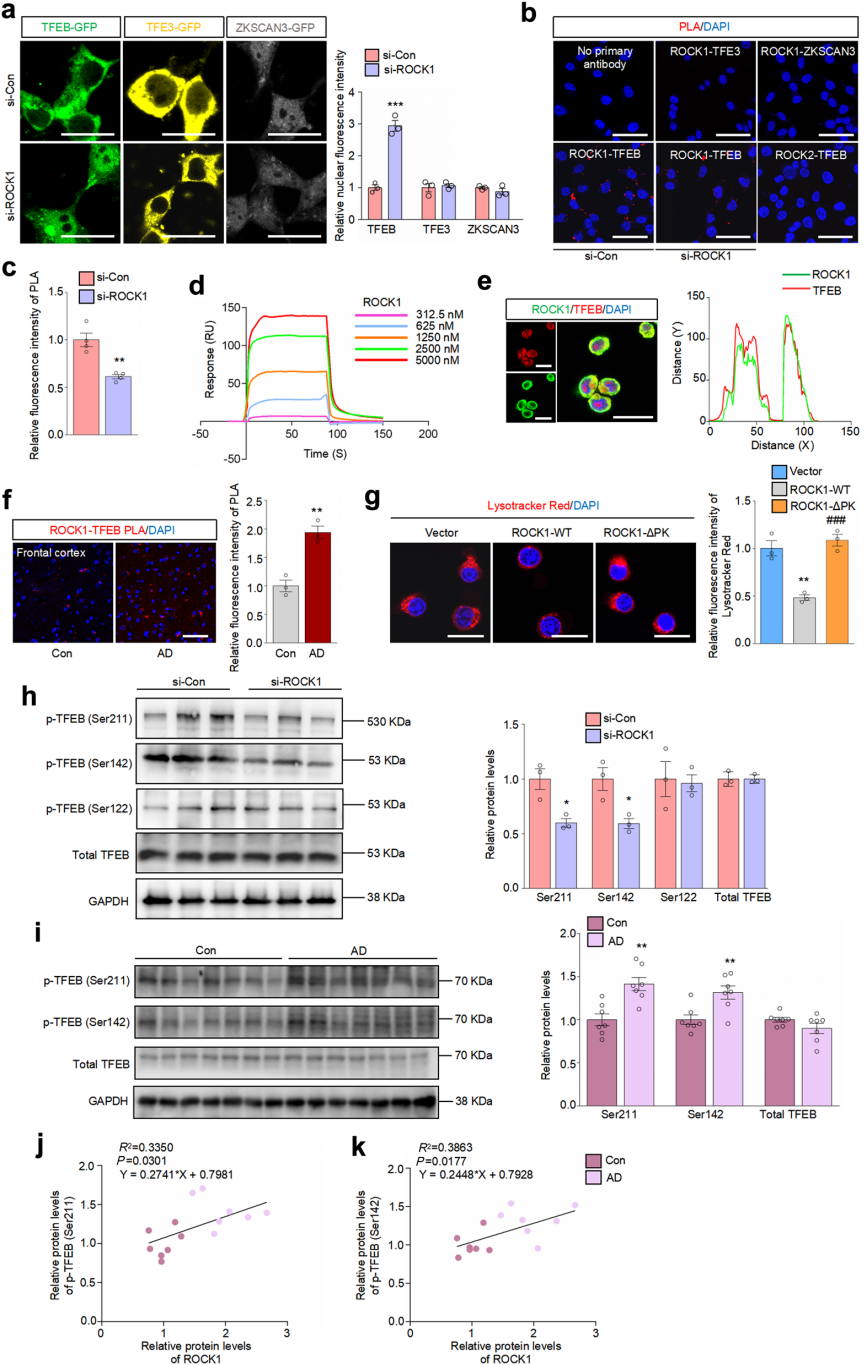
ROCK1 directly binds to TFEB and phosphorylates it at Ser211 and Ser142. **a** Subcellular localization of TFEB-GFP, TFE3-GFP and ZKSCAN3-GFP in HEK-293T cells transfected with 100 nM control siRNA or ROCK1 siRNA. *n* = 3. ***P < 0.001 versus si-Con group, Unpaired two-tailed Student’s *t*-test. Scale bars, 25 μm. **b**, **c** Proximity ligation assay (PLA) and quantification of indicated proteins in HEK-293T cells. *n* = 4. **P < 0.01, Student’s t-test. Scale bars, 50 μm. **d** Direct binding between ROCK1 and TFEB in surface plasmon resonance (SPR). Purified TFEB was coupled to the SPR sensor chip, and different concentrations of ROCK1 were injected over the surface. *n* = 3. **e** Confocal image showing co-localization of endogenous ROCK1 (green) and TFEB (red) in HEK-293T cells. Fluorescence intensity profiles are shown. *n* = 3. Scale bars, 25 μm. **f** PLA and quantification of ROCK1 and TFEB in the frontal cortex of healthy controls and AD patients. *n* = 3. **P < 0.01, Student’s *t*-test. Scale bar, 50 μm. **g** HEK-293T cells were transfected with WT ROCK1 and its truncated plasmids, followed by Lysotracker Red staining. *n* = 3. **P < 0.01 versus Vector group, ^###^*P* < 0.001 versus ROCK1-WT group. One-way ANOVA test followed by Tukey’s multiple comparisons. Scale bars, 25 μm. **h** Immunoblotting analysis and quantification of different phosphorylated-TFEBs in HEK-293T cells transfected with si-Con/si-ROCK1. *n* = 3. *P < 0.05 versus si-Con group, Student’s *t*-test. **i** Immunoblotting analysis and quantification of phosphorylated-TFEBs in the temporal cortex of healthy controls and AD patients. *n* = 7. **P < 0.01 versus Con group, Student’s *t*-test. **j**, **k** Scatter plots of the protein levels of ROCK1 and p-TFEB (Ser211) (**j**) or p-TFEB (Ser142) (**k**) in the temporal cortex of healthy controls and AD patients. Data were analyzed by a linear regression method. *n* = 7

**Fig. 5:**
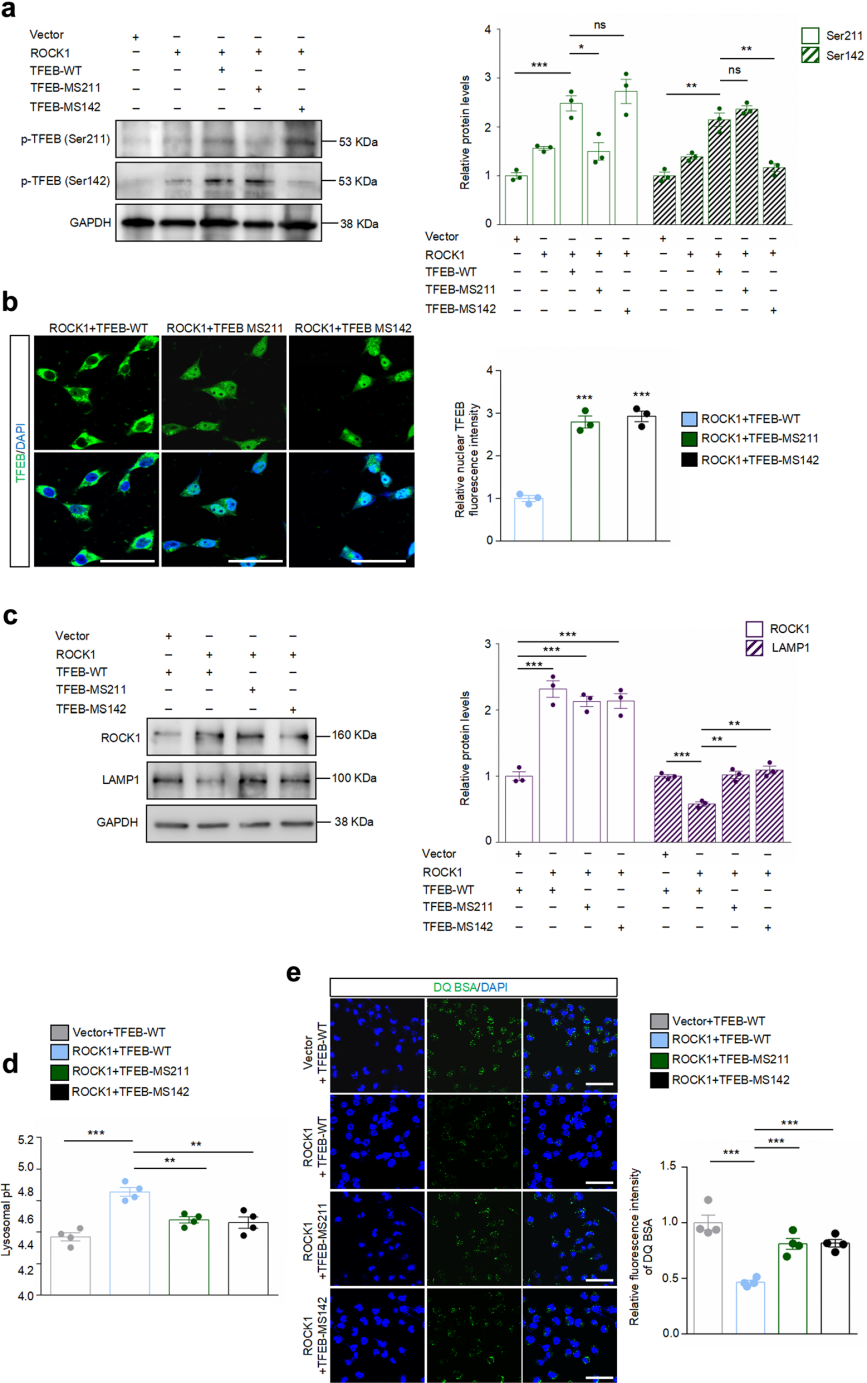
ROCK1 regulates lysosomal biogenesis, acidification, and degradation ability by phosphorylating TFEB at Ser211 and Ser142. **a** HEK-293T cells were transfected with indicated plasmids, and immunoblotting was carried out for p-TFEB (Ser211) and p-TFEB (Ser142). *n* = 3. *P < 0.05, **P < 0.01, ***P < 0.001. Statistical comparison was done using One-way ANOVA test followed by Tukey’s multiple comparisons. **b** Confocal images showing the localization of TFEB (green) in HEK-293T cells transfected with indicated plasmids. Relative nuclear TFEB fluorescence intensity was analyzed. *n* = 3. ***P < 0.001 versus ROCK1 + WT-TFEB group. Scale bar, 50 μm. **c** Immunoblotting analysis and quantification of ROCK1 and LAMP1 in HEK-293T cells transfected with indicated plasmids. *n* = 3. **P < 0.01, ***P < 0.001. **d** Lysosomal pH of HEK-293T cells transfected with indicated plasmids was determined by LysoSensor. *n* = 4. **P < 0.01, ***P < 0.001. **e** Degradation of lysosome-loaded DQ BSA in HEK-293T cells transfected with indicated plasmids. Relative fluorescence intensity of DQ BSA was analyzed. *n* = 4. ***P < 0.001. Scale bar, 50 μm. All data were analyzed with one-way ANOVA test followed by Tukey’s multiple comparisons

Lysosome biogenesis and functions can be activated by the transcription factors TFEB and/or TFE3, and repressed by ZKSCAN3 through regulating the expression of lysosomal and autophagy genes at the transcriptional level [33]. Our finding that the mRNA transcripts of lysosome-related proteins were changed upon ROCK1 overexpression or knockdown (Figs. 2b, 3b) raises the possibility that ROCK1 may modulate lysosomal biogenesis and acidification through targeting one or several transcription factors. We first assessed the influence of ROCK1 knockdown on the mRNA expression of the three transcriptional factors, none of which were affected by ROCK1 knockdown (Fig. S5a). We further evaluated the effect of ROCK1 on the nuclear-cytoplasmic trafficking of these factors. Knockdown of ROCK1 only promoted the nuclear translocation of TFEB, but did not influence the nuclear-cytoplasmic trafficking of TFE3 or ZKSCAN3 (Fig. 4a). To confirm this, cytoplasmic and nuclear fractions were obtained (Fig. S5b), and immunoblotting results showed that ROCK1 inhibition enhanced TFEB nuclear translocation (Fig. S5c, d). Consistently, endogenous TFEB was mainly localized in the cytoplasm of control cells, while more nuclear TFEB was observed after ROCK1 siRNA transfection (Fig. 5e). Nuclear translocation of TFEB is known to activate the transcription of lysosomal genes through binding CLEAR elements. Therefore, mRNA expression of several known TFEB downstream lysosome-related targets, such as ARSB, GALNS, SCPEP1, HEXA, SGSH, SQSTM1, ATP6V0D1 and ATP6V1B2, was detected and shown to be upregulated (Fig. S5f). The protein levels of SQSTM1, ATP6V0D1 and ATP6V1B2 were affected by ROCK1 downregulation as well (Fig. S5g). Downregulation of TFEB, rather than TFE3 or ZKSCAN3, prevented the si-ROCK1-induced increase of Lysotracker Red staining (Fig. S5h). The above findings indicated that ROCK1 regulates TFEB nuclear translocation and downstream targets.

To unravel the underlying mechanism by which ROCK1 regulates TFEB, we focused on m-TOR and GSK-3β, two kinases that are important for the cellular distribution and activation of TFEB [26, 34]. Importantly, neither m-TOR inhibitor Torin1, nor GSK-3β inhibitor SB415286 was able to translocate TFEB into the nucleus in ROCK1-overexpressing cells (Fig. S6a). In addition, downregulation of ROCK1 did not affect the expression of some known targets of m-TOR, including 4EBP1, S6K and ULK1 (Fig. S6b). These results suggested that the role of ROCK1 in TFEB translocation is not mainly dependent on m-TOR or GSK-3β.

We next evaluated the endogenous interaction between ROCK1 and TFEB via PLA. Our data showed that ROCK1 had a direct interaction with TFEB, but not TFE3 or ZKSCAN3 (Fig. 4b). ROCK1 knockdown caused a significant decrease in the ROCK1-TFEB binding (Fig. 4b, c). In addition, ROCK2 did not interact with TFEB directly (Fig. 4b). SPR was carried out to validate the molecular interaction between ROCK1 and TFEB in vitro, and the binding affinity *K*_d_ value of ROCK1-TFEB was confirmed to be 1.614 × 10^–6^ M (Fig. 4d, Fig. S6c). Furthermore, ROCK1 strongly co-localized with TFEB (Fig. 4e). The interaction between ROCK1 and TFEB was validated in the brains of non-demented controls, and markedly increased in the brains of AD patients (Fig. 4f). The above data collectively indicated that ROCK1 binds directly to TFEB protein.

ROCK1 serves as a vital serine-threonine kinase and phosphorylates various substrates, thus we explored whether the effects of ROCK1 on TFEB and lysosomes are dependent on its protein kinase activity. ROCK1 full-length (ROCK1-WT) and truncated plasmids (ROCK1-ΔPK, lacking the protein kinase domain and the AGC-kinase C-terminal) were constructed and introduced into HEK-293T cells (Fig. S6d). ROCK1-ΔPK transfection failed to decrease the Lysotracker Red staining as WT ROCK1 (Fig. 4g), suggesting that the protein kinase activity of ROCK1 is indispensable for regulating lysosomal activity. As ROCK1 is mainly localized in the cytoplasm, we focused on the phosphorylation sites Ser142, Ser211 and Ser122 that facilitate the cytoplasmic retention of TFEB (Fig. S6e). Knockdown of ROCK1 decreased the protein levels of phosphorylated TFEB Ser211 (p-TFEB Ser211) and TFEB Ser142 (p-TFEB Ser142), rather than phosphorylated Ser122 (p-TFEB Ser122) (Fig. 4h). Importantly, abnormal accumulation of p-TFEB Ser211 and p-TFEB Ser142 was detected in the brains of AD patients (Fig. 4i), and the protein level of ROCK1 was positively correlated with both p-TFEB Ser211 and p-TFEB Ser142 levels in human brains (Fig. 4j, k). These results indicated that the specific serine residues of TFEB (Ser211 and Ser142) are modulated by ROCK1.

### ROCK1 regulates lysosomal biogenesis, acidification, and proteolysis through targeting TFEB

We next investigated whether the regulatory role of ROCK1 on lysosomes is TFEB-dependent. TFEB WT, Ser211-mutant and Ser142-mutant plasmids were constructed (Fig. 5a) and immunofluorescence analysis revealed that the mutation at Ser211 and Ser142 largely promoted TFEB nuclear distribution in ROCK1-overexpressing cells (Fig. 5b). Notably, ROCK1 overexpression decreased LAMP1 protein level, and this effect could be abolished by both TFEB Ser211 and Ser142 mutations (Fig. 5c). Similarly, the increased expression of LAMP1 induced by ROCK1 knockdown was reversed by TFEB downregulation (Fig. S7a). The above results collectively demonstrated that ROCK1 governed lysosomal biogenesis through regulating TFEB phosphorylation.

We further confirmed the role of the ROCK1-TFEB regulatory axis in controlling lysosomal acidification and degradative ability. ROCK1 transfection impaired lysosomal acid environment and decreased proteolysis. The TFEB Ser211 mutant and Ser142 mutant, but not TFEB WT, counteracted the effects of ROCK1 transfection (Fig. 5d, e). Moreover, TFEB knockdown or lysosome inhibitor Bafilomycin A1 significantly relieved the decreased pH value and the increased proteolysis caused by ROCK1 downregulation (Fig. S7b, c). Collectively, our results demonstrated that phosphorylation of TFEB at Ser211 and Ser142 by ROCK1 is essential for the regulation of lysosomal biogenesis, acid environment and proteolytic ability.

### Aβ enhances the expression of ROCK1 and phosphorylates TFEB to impair lysosomal function

We next investigated how ROCK1 is activated in AD pathologies. Previous studies have shown that the RhoA/ROCK signaling pathway can be triggered by receptor activation under pathological conditions [35, 36], and some of these receptors are known to bind to Aβ [37]. Here we found that Aβ_42_ oligomers significantly increased ROCK1 expression and TFEB phosphorylation in both primary mouse neurons and microglia (Fig. S8a, b). Nogo receptor (NgR), a receptor of myelin-associated inhibitory factors, also acts as an Aβ receptor mediating the inhibition of synaptic assembly and plasticity [38, 39]. Moreover, NgR agonists exert physiological functions through the RhoA/ROCK pathway [40, 41]. We therefore detected whether Aβ_42_ oligomers were able to activate ROCK1 through Aβ receptor. Our data showed that the increases of ROCK1 expression and TFEB phosphorylation by Aβ could be largely reversed by two independent siRNAs targeting NgR in both neurons and microglia (Fig. S8c, d). Taken together, the above results showed that extracellular Aβ might trigger ROCK1 activation through its receptor on the surface of neural cells.

### Dampening the increase of ROCK1 promotes TFEB nuclear distribution and lysosomal function in the brains of APP/PS1 mice

**Fig. 6:**
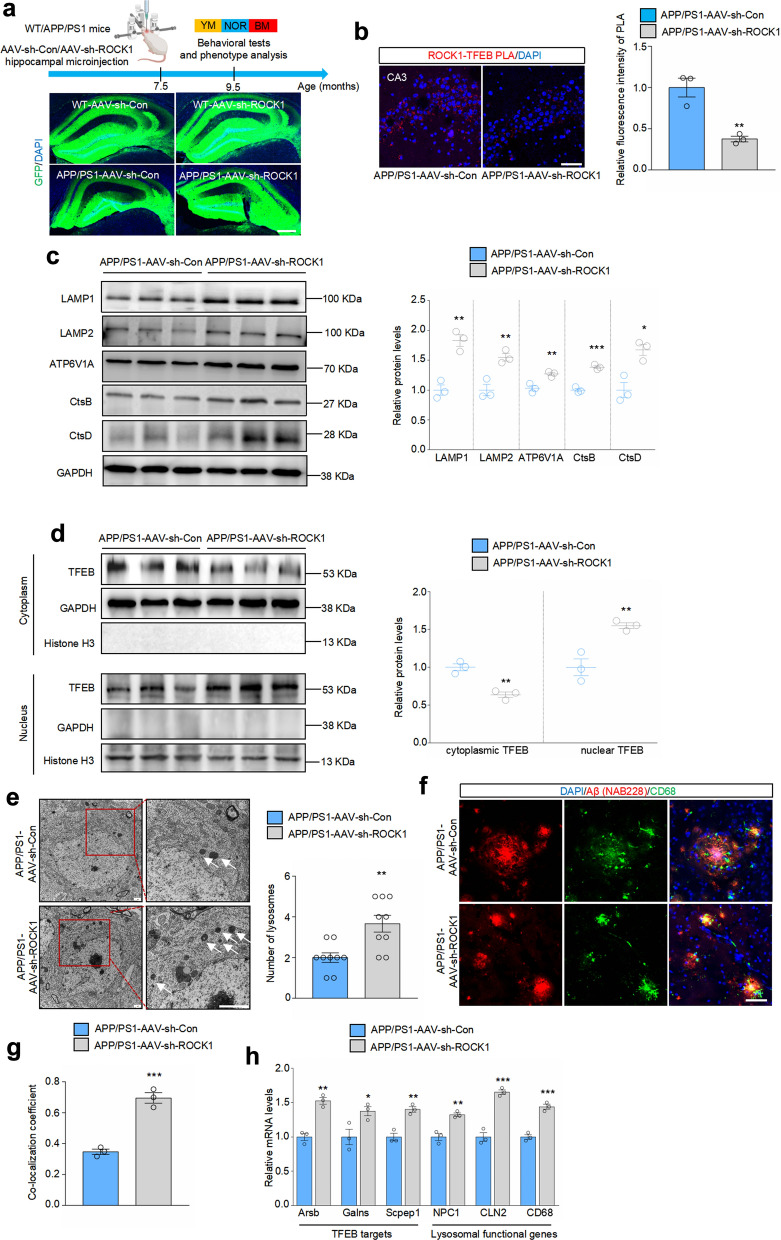
Downregulation of ROCK1 promotes TFEB nuclear translocation and lysosomal biogenesis in the brains of APP/PS1 mice. **a** Timeline of experimental procedure and confocal images of brain sections microinjected with adeno-associated virus carrying control shRNA (AAV-sh-Con) or ROCK1 shRNA (AAV-sh-ROCK1). GFP (green) was used to visualize viral diffusion. Scale bar, 200 μm. **b** PLA and quantification of ROCK1 and TFEB in the hippocampal CA3 region of APP/PS1 mice microinjected with AAV-sh-Con/AAV-sh-ROCK1. *n* = 3. Scale bar, 50 μm. **c** Immunoblotting analysis and quantification of indicated proteins in the hippocampus of APP/PS1 mice microinjected with AAV-sh-Con/AAV-sh-ROCK1. *n* = 3. **d** Immunoblotting of cytoplasmic and nuclear TFEB. *n* = 3. **e** Transmission electron microscopy of lysosomes in the hippocampus. Number of lysosomes was analyzed. *n* = 9 slices from 3 mice. Scale bar, 2 μm. White arrows indicate lysosomes. **f**, **g** Immunostaining of Aβ (red) and CD68 (green) in the hippocampus of APP/PS1 mice microinjected with AAV-sh-Con/AAV-sh-ROCK1, and co-localization coefficient for Aβ and CD68. *n* = 3. Scale bar, 50 μm. **h** qRT-PCR analysis of expression of indicated mRNAs. *n* = 3. *P < 0.05, **P < 0.01, ***P < 0.001 versus APP/PS1-AAV-sh-Con group, Student’s* t*-test

We further determined the role of ROCK1 on TFEB cellular distribution and lysosomal function in vivo. Adeno-associated virus carrying either control shRNA with GFP (AAV-sh-Con) or ROCK1 shRNA with GFP (AAV-sh-ROCK1) was microinjected into the hippocampus of 7.5-month-old WT and APP/PS1 mice (Fig. 6a). After 2 months, marked green fluorescence was observed and ROCK1 protein level was decreased in the hippocampus, indicating effective interference (Fig. 6a, Fig. S9a). PLA and nucleocytoplasmic separation-immunoblotting analysis showed that ROCK1 knockdown led to largely reduced interaction with TFEB (Fig. 6b) and increased shuttling of TFEB from the cytoplasm to the nucleus in the hippocampus of APP/PS1 mice (Fig. 6d). As expected, the nuclear translocation of TFEB caused by ROCK1 downregulation induced a strong increase of lysosomal biogenesis and function (Fig. 6c). In addition, transmission electron microscopy revealed more lysosomes in the hippocampal sections of APP/PS1 mice microinjected with AAV-sh-ROCK1 (Fig. 6e). To further examine whether increased lysosomes in ROCK1-knockdown brains could degrade toxic Aβ with higher efficiency, we co-immunostained Aβ and lysosomal marker CD68. The results showed improved co-localization of lysosomes with Aβ in the brains of AAV-sh-ROCK1 APP/PS1 mice (Fig. 6f, g). Consistently, ROCK1 knockdown led to an elevation of mRNA expression of TFEB targets and lysosomal functional genes (Fig. 6h). Taken together, we validated that ROCK1 down-regulation promotes TFEB nuclear distribution and lysosomal function in the brains of APP/PS1 mice.

### Downregulation of ROCK1 improves AD pathology and ameliorates cognitive dysfunction in APP/PS1 mice

**Fig. 7:**
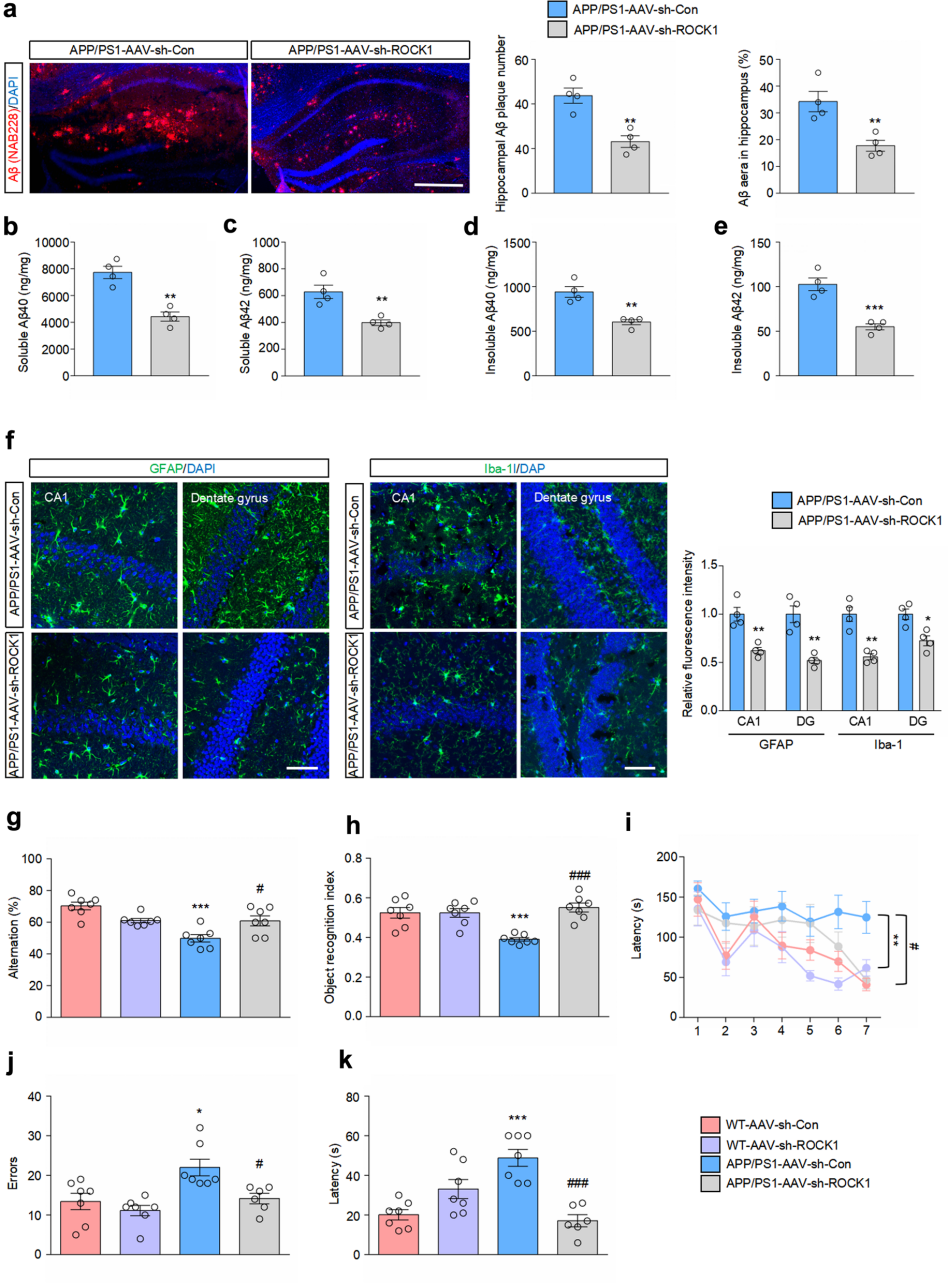
Knockdown of ROCK1 improves AD pathology and ameliorates cognitive decline in APP/PS1 mice. **a** Confocal images showing DAPI (blue) for nuclei and NAB228 (red) for Aβ in the hippocampus of APP/PS1 mice microinjected with AAV-sh-Con/AAV-sh-ROCK1. *n* = 4. Scale bar, 200 μm. **b**–**e** ELISA analysis of levels of soluble Aβ_40_ (**b**), soluble Aβ_42_ (**c**), insoluble Aβ_40_ (**d**) and insoluble Aβ_42_ (**e**) in the hippocampus of APP/PS1 mice microinjected with AAV-sh-Con or AAV-sh-ROCK1. *n* = 4. **f** Representative immunofluorescence images and quantification of GFAP (left) and Iba-1 (right) in the hippocampus. *n* = 4. Scale bars, 50 μm. **g** Y maze spontaneous alternation after microinjection with AAV-sh-Con/AAV-sh-ROCK1. *n* = 7. **h** Object recognition index in the NOR test. *n* = 7. **i** Escape latency in the Barnes maze during the training phase. *n* = 7. **j**, **k** Errors (**j**) and escape latency (**k**) in the Barnes maze trials. *n* = 6–7. **a**–**f**, *P < 0.05, **P < 0.01, ***P < 0.001 versus APP/PS1-AAV-sh-Con group, Student’s *t*-test. **g**–**k**, *P < 0.05, **P < 0.01, ***P < 0.001 vs WT-AAV-sh-Con group, ^#^*P* < 0.05, ^###^*P* < 0.001 versus APP/PS1-AAV-sh-Con group. Two-way ANOVA test followed by Tukey’s comparisons

Since downregulation of ROCK1 improved lysosomal function in APP/PS1 mice, we further investigated whether AD pathology could be improved correspondingly. We observed a significant reduction of Aβ burden in the hippocampus of APP/PS1 mice injected with AAV-sh-ROCK1 (Fig. 7a). In addition, the amyloidogenic processing of APP was inhibited and hippocampal soluble and insoluble Aβ_40_ and Aβ_42_ were decreased in the brains of APP/PS1 mice microinjected with AAV-sh-ROCK1 (Fig. 7b–e, Fig. S10). Abnormal activation of glial cells is closely related to Aβ pathology and also a pathological hallmark in AD brains [42]. We found that knockdown of ROCK1 inhibited the activation of astrocytes and microglia (Fig. 7f).

Finally, we determined whether knockdown of ROCK1 could ameliorate the cognitive deficits of APP/PS1 mice. In the Y-maze test, spontaneous alternation was obviously declined in APP/PS1 mice compared with WT mice, while this decrease was largely reversed by ROCK1 downregulation (Fig. 7g). Similarly, in the NOR test the object recognition index of the APP/PS1 mice was obviously lower than that of the WT mice, whereas ROCK1 inhibition significantly promoted the recognition memory of APP/PS1 mice (Fig. 7h). We also carried out the Barnes Maze test to assess spatial learning and long-term memory of mice. During the 7-day training phase, APP/PS1 mice spent more time finding the escape box, while ROCK1 downregulation decreased the escape latency (Fig. 7i). In the probe trials, knockdown of ROCK1 significantly reduced the number of errors and the escape latency of APP/PS1 mice (Fig. 7j, k). Taken together, our results demonstrated that ROCK1 knockdown prevented cognitive decline in APP/PS1 mice.

## Discussion

The crucial role of lysosomes in the cerebral ALP homeostasis places them at the crossroad of multiple intracellular processes such as cellular waste degradation and neuroinflammation. Thus, identifying key factors causing lysosomal dysfunction and developing lysosome-based intervention strategies have attracted growing attention for AD treatment. The present study provides new evidence indicating ROCK1, whose expression was upregulated in the brains of AD mouse models and human postmortem cortical samples, as a potential culprit of lysosomal dysfunction. Knockdown of ROCK1 promoted lysosomal biogenesis and acidification in the lysosomal lumen, ameliorating proteotoxicity and AD-related pathology. Mechanistically, our results demonstrated that ROCK1 regulated lysosomal function specifically through TFEB, a transcription factor that boosts expression of lysosome genes. ROCK1 downregulation relieved the phosphorylation of TFEB, which in turn promoted TFEB nuclear translocation and induced the transcription of CLEAR genes. In vivo, we proved that the AAV-mediated ROCK1 interference could promote Aβ clearance and improve cognitive function in APP/PS1 AD model mice by targeting lysosomes.

Our results indicated that ROCK1 levels were increased in AD brains and correlated with lysosomal and autophagic markers, and the lysosomes in ROCK1-overexpressed cells, similar to those observed in AD, showed significant dysfunction of biogenesis and acidification. These results suggested that the elevated expression and activity of ROCK1 may be an important cause of lysosomal dysfunction, which induces a domino effect that eventually compromises neurodegeneration. Previous studies have shown that ROCK2 is mainly expressed in neurons while ROCK1 is distributed in glial cells [43, 44]. In this work, we also identified expression of ROCK1 in neurons and found that ROCK1 downregulation maintained lysosomal homeostasis in both neurons and microglia. In addition, we showed that Aβ activated ROCK1 and enhanced TFEB phosphorylation, which further hindered the ALP-mediated Aβ clearance in both neurons and microglia, thus forming a deleterious cycle. This effect was largely mediated by Aβ receptor NgR, which is present on both types of cells. Our study provides a novel pattern of Aβ activation of cellular ROCK1, although it is unclear if this is the only pathway by which Aβ activates cellular ROCK1.

To meet the need of cellular metabolite recycling and signal transduction, cells increase lysosome numbers and volumes by inducing the transcription of lysosomal genes, a process highly regulated by transcription factors such as TFEB, TFE3 and ZKSCAN3 [33]. Having clarified that ROCK1 does not regulate lysosome production through the endosomal delivery pathway, we comprehensively investigated the effect of ROCK1 on these key factors, and showed that TFEB was a core downstream mediator of ROCK1. TFEB is a basic helix-loop-helix leucine zipper transcription factor that binds to the promoters of CLEAR genes and therefore promotes lysosomal biogenesis and function [45–47]. Phosphorylation of TFEB traps itself in the cytoplasm and reduces its nuclear translocation, thus preventing the binding of TFEB to the promoters of lysosome-related genes [48–50]. Therefore, reduction of TFEB activity or expression directly contributes to impaired ALP and age-related neurodegeneration. Conversely, a direct increase in TFEB level or nuclear localization alleviates Aβ and tau pathology through benefiting lysosomal biogenesis [51–54]. Here we reported novel serine-specific phosphorylation of TFEB by ROCK1 in AD pathology, which resulted in TFEB inactivation without influencing its expression. However, ROCK1 was not found to specifically bind to or regulate TFE3 or ZKSCAN3, indicating that the lysosome-associated transcription factors are regulated through distinct mechanisms.

m-TOR and GSK-3β are regarded as critical regulators of TFEB and lysosomal adaptation to deal with nutrient supply and starvation [26–28]. Our research uncovered ROCK1 as an endogenous repressor of TFEB in AD without compromising m-TOR and GSK-3β, providing novel insights into different lysosomal regulation pathways in response to various environmental cues. ROCK1 has been reported to phosphorylate GSK-3β at Tyr216 to activate it in neurons [55], implying that GSK-3β phosphorylation, which leads to inhibition of TFEB nuclear translocation, may mediate in part the regulatory effect of ROCK1 on TFEB. However, our current results that ROCK1 still trapped TFEB in the cytoplasm upon GSK-3β inhibition indicated that ROCK1 regulated TFEB nuclear translocation mainly through a direct phosphorylation manner. In addition, although TFEB is able to drive endocytosis [56], endocytosis inhibitors cannot prevent the effect of ROCK1 on lysosomal biogenesis, suggesting that ROCK1 regulates lysosomal function mainly through TFEB-mediated expression of CLEAR genes.

Besides manipulating lysosomal biogenesis, TFEB can also impact lysosomal acidification by transcriptionally activating V-ATPase subunits [57]. An acidic internal environment in mature lysosomes is critical for lysosomal hydrolases to exert biological function [58]. Maintenance of the acidic environment in lysosomes depends on the coordination of proton pumps on the membrane. V-ATPase is a type of H^+^ pump widely expressed in the vacuolar membrane system of eukaryotic cells, which is composed of transmembrane V0 and intracellular V1 subunits. When these two subunits polymerize, the energy generated by ATP decomposition can be used to transport H^+^ against the concentration gradient, thus maintaining a relatively acidic pH inside the lysosomes [59, 60]. In this regard, we showed that ROCK1 could regulate the lysosomal acidic environment through regulating V1 subunit expression and V-ATPase assembly via TFEB.

After confirming the critical roles of ROCK1 in lysosomal biogenesis and function, we further explored the effect of ROCK1 manipulation on AD pathology and cognitive decline. Since most ROCK inhibitors such as Y-27632 are nonselective to ROCK1 and ROCK2, we performed ROCK1-specific RNA interference in mice. Our data suggested that ROCK1 knockdown decreased Aβ burden, at least partly due to the increased lysosomal numbers and functions, although ROCK1 has been reported to affect Aβ levels by acting on APP processing [61]. In the brains of APP/PS1 mice, glial cells are hyperactivated, with enlarged footprints of reactive glial nets and increased neuroinflammation but insufficient access to Aβ deposits, leading to reduced Aβ phagocytosis and clearance. ROCK1 downregulation in microglia promoted their lysosomal function and reduced their activation to an intermediate state, and eventually ameliorated the cognitive impairment of APP/PS1 mice.

## Conclusion

In summary, our study provided for the first time solid evidence demonstrating that ROCK1 is involved in the regulation of lysosomes by modulating TFEB phosphorylation and nuclear-cytoplasmic trafficking, and consequently influences Aβ clearance, neuronal survival and cognitive function in AD pathogenesis. The findings advance our understanding of ROCK1 function in lysosomal regulation and provide insights into the treatment of AD and other neurodegenerative diseases concerning lysosomal dysfunction.

## Supplementary Information

Additional file 1: **Table S1.** Demographics of human brain donors for the immunoblotting experiment. **Table S2**. Demographics of human brain donors for immunostaining and PLA experiments. **Table S3**. siRNAs used in this study. **Table S4**. Primer sets used for qRT-PCR.

Additional file 2: **Figure S1** ROCK1 is increased in AD brains. **Figure S2** Downregulation of ROCK1 promotes lysosomal function in primary microglia. **Figure S3** ROCK1 knockdown increases lysosomal numbers and maintains lysosomal acid environment in cell lines. **Figure S4** The regulatory role of ROCK1 on lysosomal biogenesis is independent of endosome-lysosome pathway. **Figure S5** ROCK1 downregulation increases the nuclear localization of TFEB. **Figure S6 **The regulatory role of ROCK1 on TFEB is not mainly dependent on m-TOR and GSK-3β. **Figure S7** Downregulation of TFEB attenuates the regulatory effects of ROCK1 downregulation on lysosome. **Figure S8** Aβ increases ROCK1 and phosphorylated TFEB levels to impair lysosomal function. **Figure S9** Knockdown efficiency of ROCK1 shRNA in the hippocampus of WT and APP/PS1 mice. **Figure S10** ROCK1 downregulation inhibits amyloidogenic processing of APP in the brain of APP/PS1 mice.

## Data Availability

All data generated or analysed during this study are included in this published article and its supplementary files.

## Abbreviations

ROCK1: Rho-associated coiled-coil kinase 1
ALP: Autophagy-lysosomal pathway
AD: Alzheimer’s disease
TFEB: Transcription factor EB
V-ATPase: Vacuolar-type proton adenosine triphosphatase
LAMP: Lysosome-associated membrane glycoprotein
CtsB: Cathepsin B
CtsD: Cathepsin D
M6PR: Mannose-6-phosphate receptor
NgR: Nogo receptor
