# Supplementary material for: Critical role of ROCK1 in AD pathogenesis via controlling lysosomal biogenesis and acidification
Source: Transl Neurodegener. 2024 Nov 4;13:54. doi: 10.1186/s40035-024-00442-9 (PMC11533276; doi:10.1186/s40035-024-00442-9)
Supplement: Supplementary file 1 — Additional file 1: Table S1. Demographics of human brain donors for the immunoblotting experiment. Table S2. Demographics of human brain donors for immunostaining and PLA experiments. Table S3. siRNAs used in this study. Table S4. Primer sets used for qRT-PCR. [file 40035_2024_442_MOESM1_ESM.docx]

**Additional file 1.**

**Table S1 Demographics of human brain donors for the immunoblotting experiment**

| **No.** | **Source** | **Age (years)** | **Sex** | **Diagnosis** | **Pmd** | **Braak** |
| --- | --- | --- | --- | --- | --- | --- |
| 1 | NBB | 60 | Female | NDC | 6 h 50 min | 1 |
| 2 | NBB | 78 | Female | NDC | 7 h 10 min | 1 |
| 3 | NBB | 76 | Female | NDC | 6 h 30 min | 1 |
| 4 | NBB | 72 | Female | NDC | 6 h 50 min | 1 |
| 5 | NBB | 96 | Male | NDC | 6 h 30 min | 1 |
| 6 | NBB | 80 | Male | NDC | 4 h 25 min | 2 |
| 7 | NBB | 82 | Male | NDC | 12 h 55 min | 3 |
| 8 | NBB | 76 | Male | AD | 5 h 10 min | 6 |
| 9 | NBB | 73 | Male | AD | 6 h 15 min | 6 |
| 10 | NBB | 73 | Male | AD | 4 h 45 min | 5 |
| 11 | NBB | 61 | Male | AD | 4 h 15 min | 5 |
| 12 | NBB | 68 | Female | AD | 3 h 50 min | 6 |
| 13 | NBB | 85 | Female | AD | 5 h | 5 |
| 14 | NBB | 79 | Male | AD | 5 h 5 min | 5 |

NBB: Netherlands Brain Bank

NDC: Non-demented control

Pmd: Post-mortem delay

**Table S2 Demographics of human brain donors for immunostaining and PLA experiments**

| **No.** | **Source** | **Age (years)** | **Sex** | **Diagnosis** | **Pmd** |
| --- | --- | --- | --- | --- | --- |
| 1 | NHBB | 76 | Male | NDC | 6 h |
| 2 | NHBB | 79 | Male | NDC | 3h 30 min |
| 3 | NHBB | 67 | Male | NDC | 5 h |
| 4 | NHBB | 80 | Female | AD | 18 h |
| 5 | NHBB | 80 | Male | AD | 4h 30 min |
| 6 | NHBB | 85 | Female | AD | 4 h 30 min |

NHBB: National Human Brain Bank for Development and Function

NDC: Non-demented control

Pmd: Post-mortem delay

**Table S3 siRNAs used in this study**

| **Oligo set** | **Sequences** |
| --- | --- |
| ROCK1 | 5'-GCGUUUGCCAAUAGUCCUUTT-3' (sense); |
|  | 5'-AAGGACUAUUGGCAAACGCTT-3' (anti-sense) |
| TFEB | 5'-CCAAGAAGGAUCUGGACUUTT-3' (sense); |
|  | 5'-AAGUCCAGAUCCUUCUUGGTT-3' (anti-sense) |
| TFE3 | 5'-AAUGACAUCAUCAAUCUCCUU-3' (sense); |
|  | 5'-GGAGAUUGAUGAUGUCAUUGA-3' (anti-sense) |
| ZKSCAN3 | 5'-ACACAUACUGCACUGAUACGG-3' (sense); |
|  | 5'-GUAUCAGUGCAGUAUGUGUGG-3' (anti-sense) |
| NgR-1 | 5'-AUCACUAAGAUCUAGUUGCUC-3' (sense); |
|  | 5'-GCAACUAGAUCUUAGUGAUAA-3' (anti-sense) |
| NgR-2 | 5'-UAUCACUAAGAUCUAGUUGCU-3' (sense); |
|  | 5'-CAACUAGAUCUUAGUGAUAAU-3' (anti-sense) |

**Table S4 Primer sets used for qRT-PCR**

| **Primer set** | **Sequences** |
| --- | --- |
| Homo ROCK1 | 5'-GAATGTGACTGGTGGTCGGT-3' (forward); |
|  | 5'-CTGGTGCTACAGTGTCTCGG-3' (reverse) |
| Mus Rock1 | 5'-AACGCTCCGAGACACTGTAG-3' (forward); |
|  | 5'-ACTTTCCTGCAAGCTTTTATCCAC-3' (reverse) |
| Homo LAMP1 | 5'-CACACCTTTTCCCCAATGCG-3' (forward); |
|  | 5'-TGTTCACAGCGTGTCTCTCC-3' (reverse) |
| Mus Lamp1 | 5'-ATGAGAAGGCTCCACTGATTTG-3' (forward); |
|  | 5'-CAGAGATTCCCTTTGTAAGGCTTAA-3' (reverse) |
| Homo LAMP2 | 5'-TGGCTCCGTTTTCAGCATTG-3' (forward); |
|  | 5'-CGCTATGGGCACAAGGAAGT-3' (reverse) |
| Mus Lamp2 | 5'-GCCCCTCTGGGAAGTTCTTA-3' (forward); |
|  | 5'-ATGGGCACAAGGAAGTTGTCT-3' (reverse) |
| Homo NPC1 | 5'-TGGAGGGATTGTGGTGTTGG-3' (forward); |
|  | 5'-ATCGCTCTTCAGTGGCACAA-3' (reverse) |
| Mus Npc1 | 5'-CCACAGAAGGCGGTACTTTG-3' (forward); |
|  | 5'-GGATTGGTGGTGACCTGGAC-3' (reverse) |
| Homo CLN2 | 5'-GCAACTTTGCACATCAGGCA-3'(forward); |
|  | 5'-GTGTTGACCCGCTGGATGTA-3' (reverse) |
| Mus Cln2 | 5'-GCCGCACTATCTGATGGCTA-3'(forward); |
|  | 5'-CCTGTCCCATGCTGCTGATA-3' (reverse) |
| Homo CtsB | 5'-CTCCTGCTGGCTGTAATGGT-3' (forward); |
|  | 5'-CTGTTTGTAGGTCGGGCTGT-3' (reverse) |
| Mus Ctsb | 5'-ATTCACACCAATGGCCGAGT-3'(forward); |
|  | 5'-CATTGACATGGTGCTCGCAG-3' (reverse) |
| Homo CtsD | 5'-GGCGAGTACATGATCCCCTG-3' (forward); |
|  | 5'-GTGTAGTAGCGGCCGATGAA-3' (reverse) |
| Mus Ctsd | 5'-CCAAGCAGCCTGGAATCGTA-3'(forward); |
|  | 5'-GCTCCCCGTGGTAGTACTTG-3' (reverse) |
| Homo ATP6V1A | 5'-AGAAGCTGCCAGCCAATCAT-3' (forward); |
|  | 5'-TCCCGGAGGACTTCAGACAT-3' (reverse) |
| Mus Atp6v1a | 5'-CATCCGATGTCGTCCTGGAG-3' (forward); |
|  | 5'-AAGGGCATCGAGGACTCTCT-3' (reverse) |
| Homo TFEB | 5'-AGGAGCGGCAGAAGAAAGAC-3' (forward); |
|  | 5'-CAGAGGCCTTGAGGATGGTG-3' (reverse) |
| Homo TFE3 | 5'-CCAGAGCAGCTGGACATTGA-3' (forward); |
|  | 5'-CAGCAGGGGACACTGAAGAG-3' (reverse) |
| Homo ZKSCAN3 | 5'-TAGAGTTCTCCAGGTCCCCG-3' (forward); |
|  | 5'-CTTTTCCAGGCCTCTCCCTG-3' (reverse) |
| Mus Cd68 | 5'-ACCTACATCAGAGCCCGAGT-3' (forward); |
|  | 5'-GAAGTGTCCCTTGTCAGGCA-3' (reverse) |
| Homo ARSB | 5'-CGGGAGCTCATCCACATCTC-3' (forward); |
|  | 5'-CACGGTGAAGAGTCCACGAA-3' (reverse) |
| Mus Arsb | 5'-CAGCTGTGTTCCCCTAGACG-3' (forward); |
|  | 5'-GGAACAGTGGTTTCTCCGGT-3' (reverse) |
| Homo GALNS | 5'-CAACATCCTGCTCCTGCTCA-3' (forward); |
|  | 5'-TGTGTAGGCGTTTCTGGCAT-3' (reverse) |
| Mus Galns | 5'-CACAACCAACGCTCATGCAA-3' (forward); |
|  | 5'-AAGATCAGGGGTAGCTCCGT-3' (reverse) |
| Homo SCPEP1 | 5'-GCCACGTGAGACACCTACAA-3' (forward); |
|  | 5'-CAGCAACTCGTCCACAATGC-3' (reverse) |
| Mus Scpep1 | 5'-GCTGCTGGCATCAGTGTAGA-3' (forward); |
|  | 5'-ACTTGCTCTGCAATGTCGGA-3' (reverse) |
| Homo HEXA | 5'-ACCAGCGCTACGTCCTTTAC-3' (forward); |
|  | 5'-GGAAGACAGGGTAAGCTTGGT-3' (reverse) |
| Homo SGSH | 5'-GCATGGACCAAGGAGTTGGA-3' (forward); |
|  | 5'-TGGGCGTGAGGTCTAGGAG-3' (reverse) |
