# Supplementary material for: Critical role of ROCK1 in AD pathogenesis via controlling lysosomal biogenesis and acidification
Source: Transl Neurodegener. 2024 Nov 4;13:54. doi: 10.1186/s40035-024-00442-9 (PMC11533276; doi:10.1186/s40035-024-00442-9)
Supplement: Supplementary file 2 — Additional file 2: Figure S1 ROCK1 is increased in AD brains. Figure S2 Downregulation of ROCK1 promotes lysosomal function in primary microglia. Figure S3 ROCK1 knockdown increases lysosomal numbers and maintains lysosomal acid environment in cell lines. Figure S4 The regulatory role of ROCK1 on lysosomal biogenesis is independent of endosome-lysosome pathway. Figure S5 ROCK1 downregulation increases the nuclear localization of TFEB. Figure S6 The regulatory role of ROCK1 on TFEB is not mainly dependent on m-TOR and GSK-3β. Figure S7 Downregulation of TFEB attenuates the regulatory effects of ROCK1 downregulation on lysosome. Figure S8 Aβ increases ROCK1 and phosphorylated TFEB levels to impair lysosomal function. Figure S9 Knockdown efficiency of ROCK1 shRNA in the hippocampus of WT and APP/PS1 mice. Figure S10 ROCK1 downregulation inhibits amyloidogenic processing of APP in the brain of APP/PS1 mice. [file 40035_2024_442_MOESM2_ESM.docx]

Additional file 2


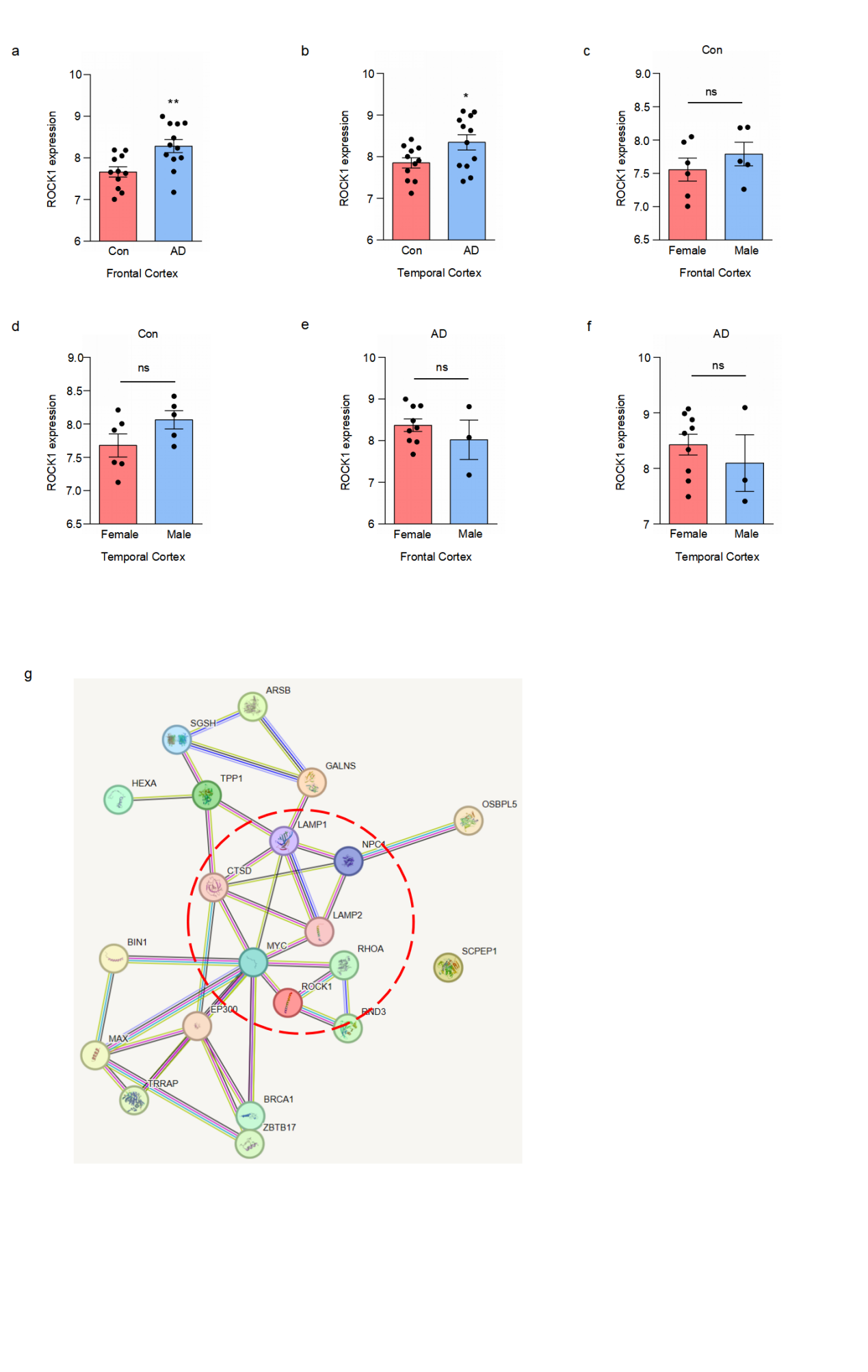


Figure S1 ROCK1 is increased in AD brains. a, b ROCK1 was increased in the frontal (a) and temporal cortex (b) of AD individuals compared with healthy controls. n(Con) = 11, n(AD) = 12. **P* < 0.05, ***P* < 0.01 vs Con group. Data from: GSE122063. c, d ROCK1 expression in the frontal (c) and temporal cortex (d) of female and male healthy controls. n(Female) = 6, n(Male) = 5. ns = no significance. Data from: GSE122063. e, f ROCK1 expression in the frontal (c) and temporal cortex (d) of female and male AD individuals. n(Female) = 9, n(Male) = 3. ns = no significance. Data from: GSE122063. g Protein-protein interaction analysis showed that ROCK1 was strongly correlated with many known lysosome-related proteins. Data supported by: <https://cn.string-db.org/>


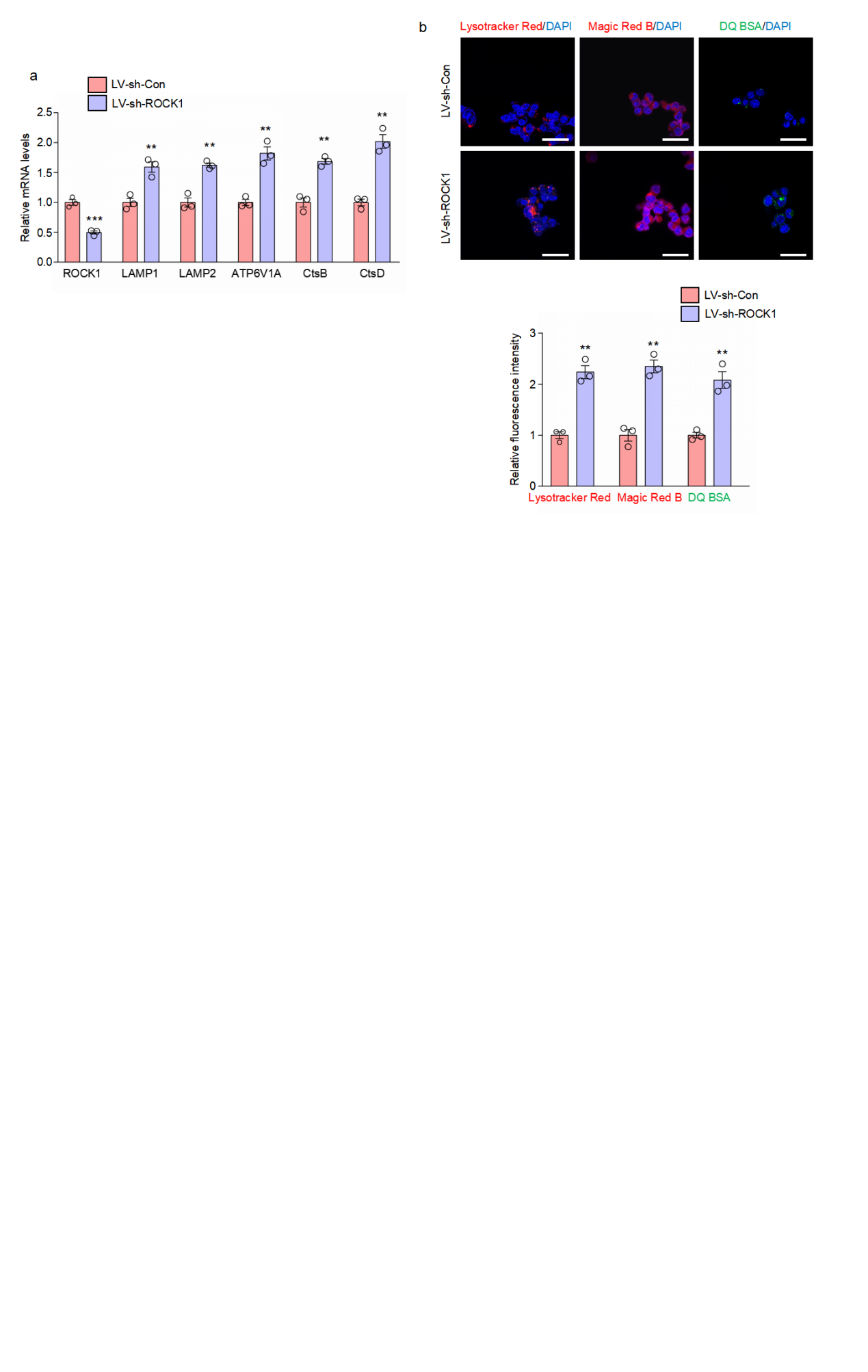


Figure S2 Downregulation of ROCK1 promotes lysosomal function in primary microglia. a Primary mouse microglia were infected with lentiviral preparations expressing either sh-Con or sh-ROCK1 for 48 h. qRT-PCR was carried out to detect lysosomal markers. *n =* 3. ***P* < 0.01, ****P* < 0.001 vs LV-sh-Con group using Student’s t-test. b Lysotracker Red, Magic Red B and DQ BSA staining in primary mouse microglia infected with LV-sh-Con/LV-sh-ROCK1. The respective fluorescence intensity was analyzed. *n =* 3. ***P* < 0.01 vs LV-sh-Con group using Student’s t-test. Scale bar, 50 μm.


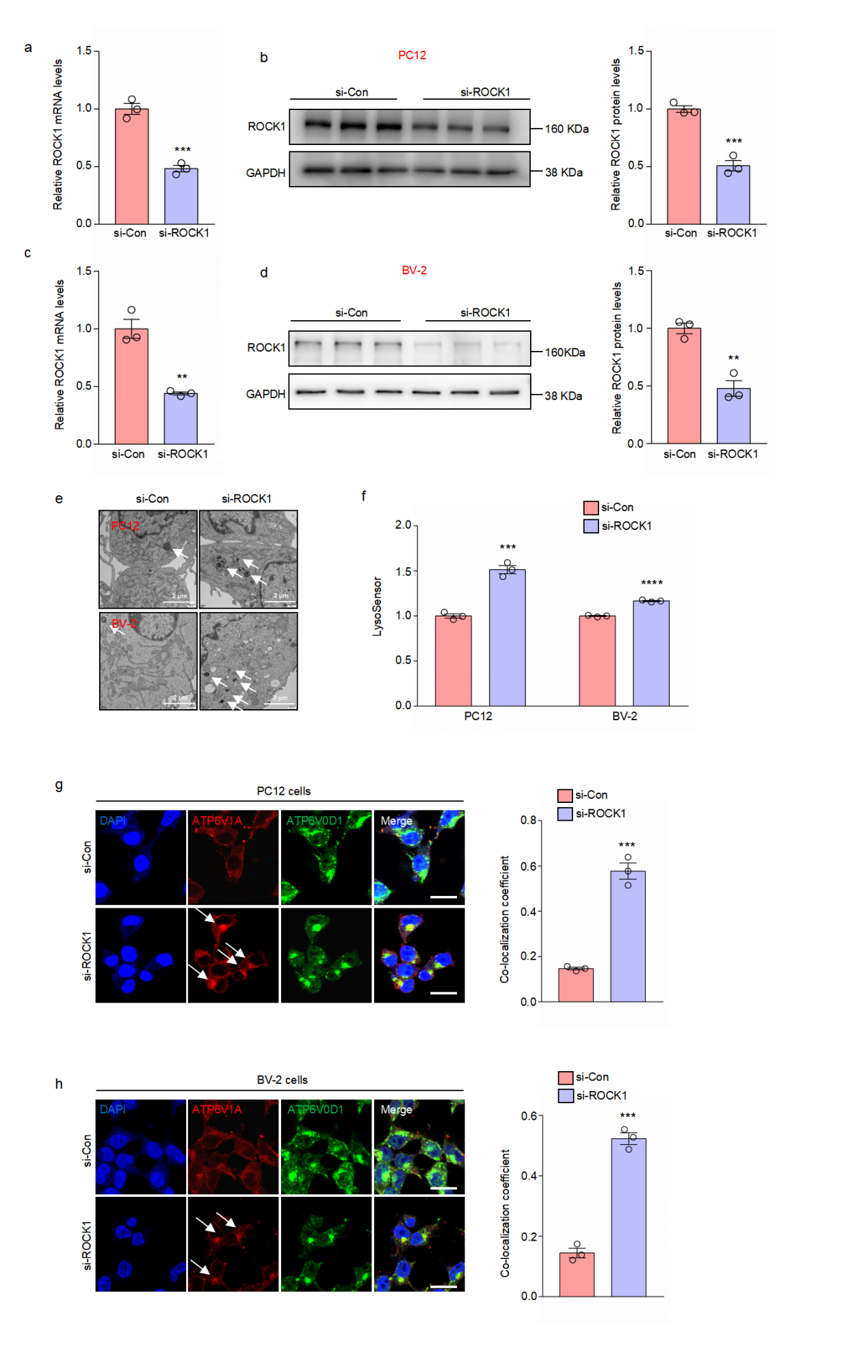


Figure S3 ROCK1 knockdown increases lysosomal numbers and maintains lysosomal acid environment in cell lines. a-b 100 nM si-Con/si-ROCK1 were transfected into PC12 cells. qRT-PCR and immunoblotting were used to detect the mRNA (a) and protein (b) levels of ROCK1. *n =* 3. ****P* < 0.001 vs si-Con group using Student’s t-test. c-d 100 nM si-Con/si-ROCK1 were transfected into BV-2 cells. qRT-PCR and immunoblotting were used to detect the mRNA (c) and protein (d) levels of ROCK1. *n =* 3. ***P* < 0.01 vs si-Con group using Student’s t-test. e Transmission electron microscopy was performed to observe lysosomes in cell lines. *n =* 3. Scale bar, 2 μm. The white arrows indicate the lysosomes. f The LysoSensor reagents were used to detect the pH value inside lysosomes in cell lines according to fluorescence intensity. *n =* 3. ****P* < 0.001, *****P* < 0.0001 vs si-Con group using Student’s t-test. g-h PC12 cells (g) and BV-2 cells (h) were transfected with indicated si-Con/si-ROCK1. Double-immunostaining for ATP6V1A (V1 component, red) and ATP6V0D1 (V0 component, green) was performed and the co-localization coefficient was analyzed. Nuclei were stained with DAPI (blue). *n =* 3. ****P* < 0.001 vs si-Con group. Scale bar, 25 μm.


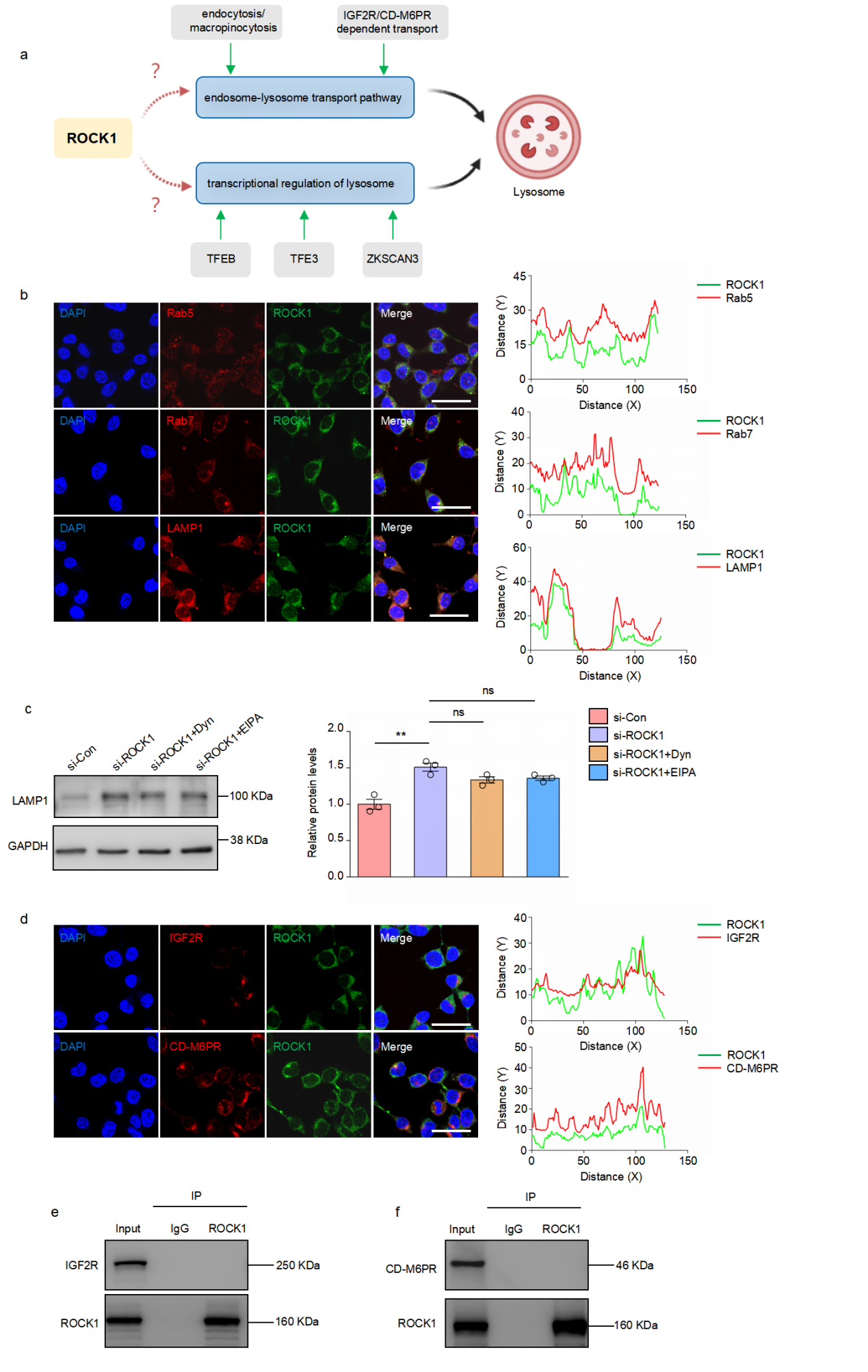


Figure S4 The regulatory role of ROCK1 on lysosomal biogenesis is independent of endosome-lysosome pathway. a Schematic diagram of possible mechanisms by which ROCK1 regulates lysosome. b Double staining of ROCK1 (green) and markers for subcellular organelles (red) in HEK-293T cells and the fluorescence intensity profiles are shown. Nuclei were stained with DAPI (blue). *n =* 3. Scale bar, 50 µm. c HEK-293T cells were pre-incubated with dynasore (DYN) or 5-(n-ethyl-n-isopropyl) amiloride (EIPA) for 1 h, and then transfected with si-Con/si-ROCK1 for 24 h. Immunoblotting was carried out to determine LAMP1 protein expression. *n =* 3. ns = no significance. ***P* < 0.01 vs indicated group. Data are analyzed with one-way ANOVA. d Double staining of ROCK1 (green) and IGF2R (red) or M6PR (red) in HEK-293T cells and the fluorescence intensity profiles are shown. Nuclei were stained with DAPI (blue). *n =* 3. Scale bar, 50 µm. e, f HEK-293T cells were immunoprecipitated (IP) with anti-ROCK1 monoclonal antibody or IgG. Immunoblotting was performed to determine IGF2R (E) or M6PR (F). *n =* 3.


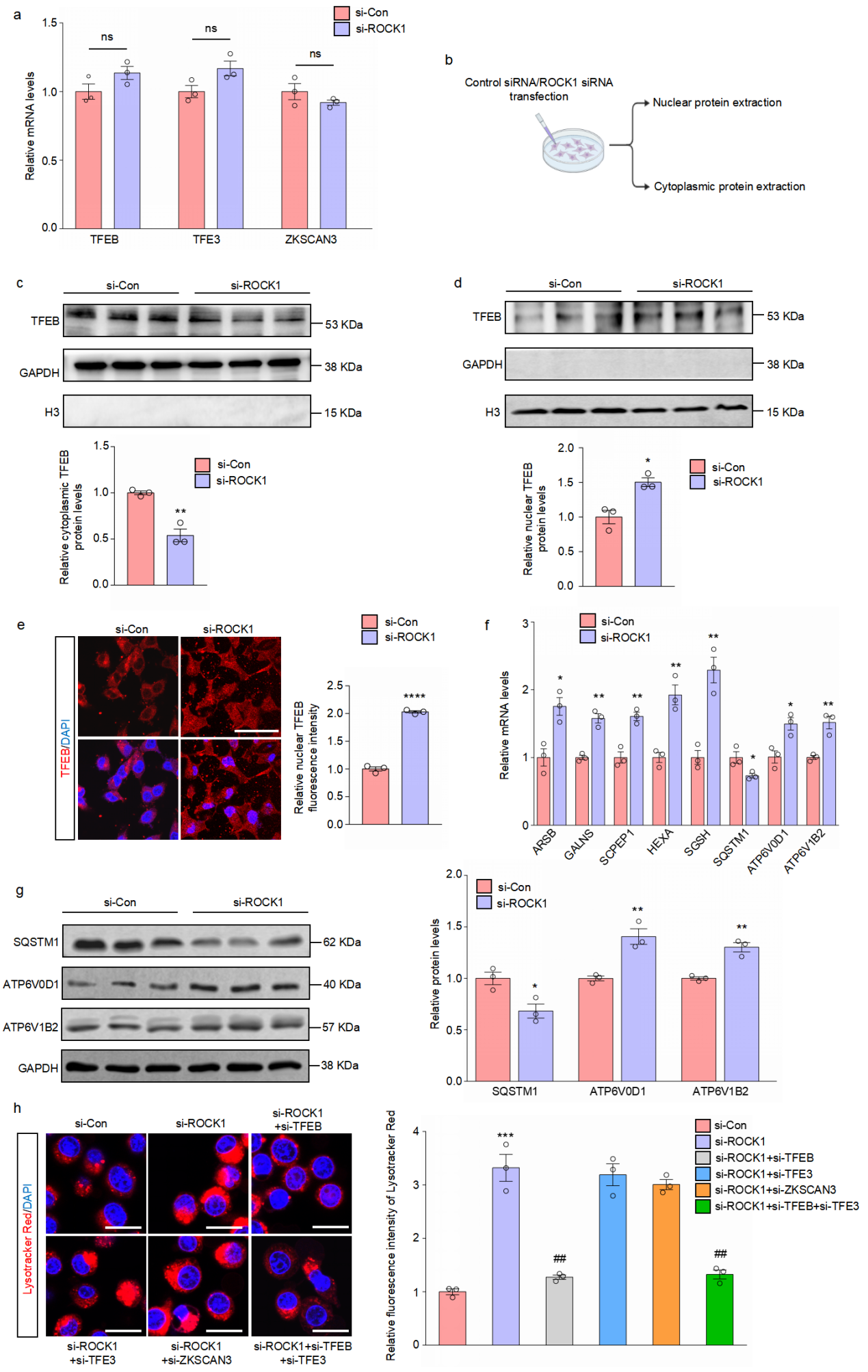


Figure S5 ROCK1 downregulation increases the nuclear localization of TFEB. a qRT PCR was performed to determine the indicated mRNA levels in HEK-293T cells transfected with si-Con/si-ROCK1. *n =* 3. ns = no significance. b Schematic illustration of the experimental procedure created with BioRender.com. c-d Cytoplasmic and nuclear proteins were separated from HEK-293T cells transfected with si-Con/si-ROCK1, and the levels of TFEB in the cytoplasm and nucleus were detected by immunoblotting respectively. *n =* 3. **P* < 0.05, ***P* < 0.01 vs si-Con group using Student’s t-test. e Confocal image showed the localization of TFEB (red) in HEK-293T cells transfected with si-Con/si-ROCK1, and the relative nuclear TFEB fluorescence intensity was analyzed. DAPI was used to stain nucleus (blue). *n =* 3. *****P* < 0.0001 vs si-Con group. Scale bar, 50 μm. f qRT-PCR analysis was used to determine the expression levels of mRNAs. *n =* 3. **P* < 0.05, ***P* < 0.01 vs si-Con group using Student’s t-test. g Immunoblotting was carried out to detect the protein levels of SQSTM1, ATP6V0D1 and ATP6V1B2 after knockdown of ROCK1 in HEK-293T cells. *n =* 3. **P* < 0.05, ***P* < 0.01 vs si-Con group. h HEK-293T cells were transfected with the indicated siRNAs for 24 h, followed by staining with Lysotracker Red. *n =* 3. ****P* < 0.001 vs si-Con group, ^##^*P* < 0.01 vs si-ROCK1 group. Scale bar, 25 μm.


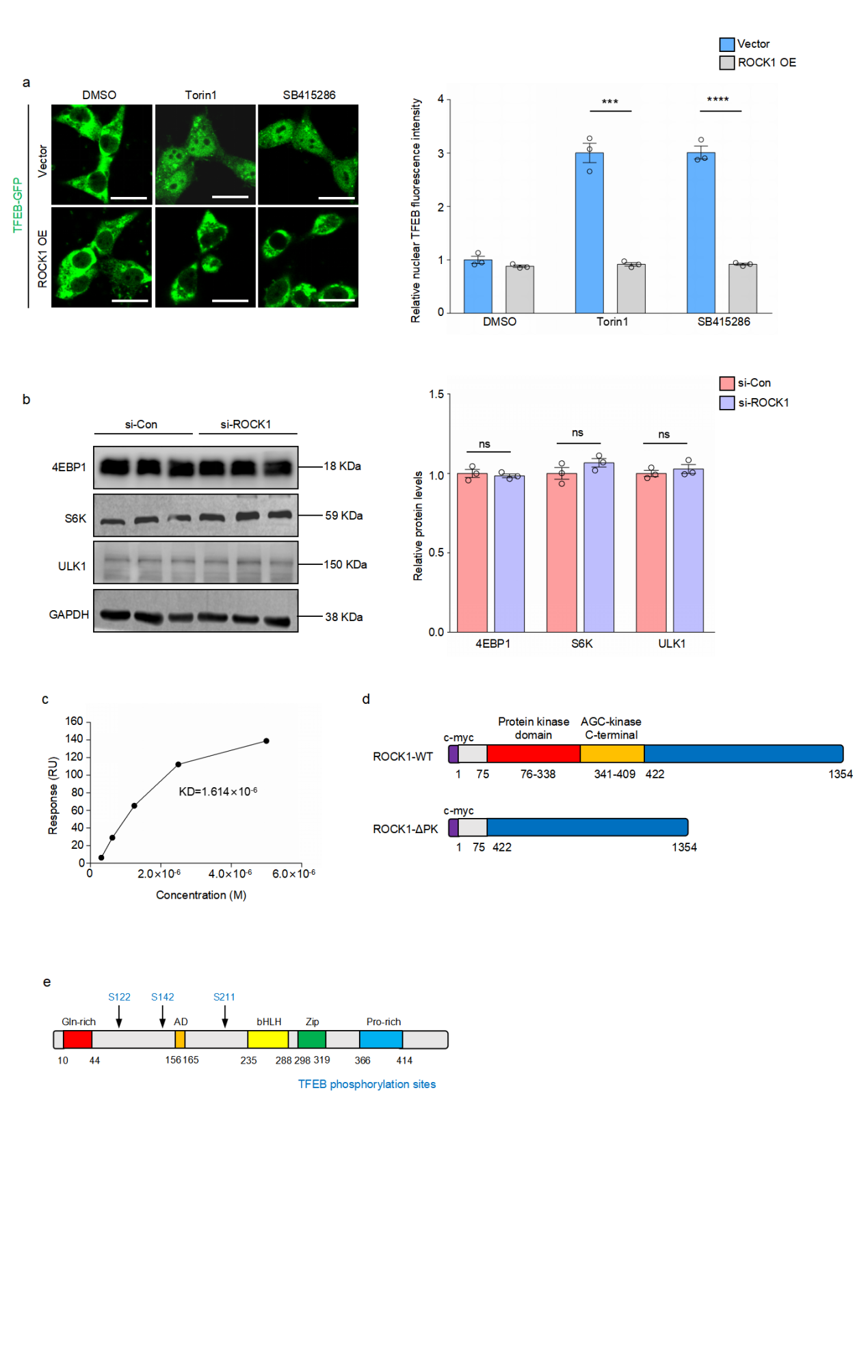


Figure S6 The regulatory role of ROCK1 on TFEB is not mainly dependent on m-TOR and GSK-3β. a HEK-293T cells transfected with ROCK1 plasmid, and treated with Torin1 or SB415286 for 2 h. *n =* 3. ****P* < 0.001, *****P* < 0.0001 vs indicated group. Scale bar, 25 μm. b HEK-293T cells were transfected with ROCK1 siRNAs, and the protein levels of 4EBP1, S6K and ULK1 were detected by immunoblotting. *n =* 3. ns = no significance. c Purified TFEB was coupled to the SPR sensor chip, and different concentrations of ROCK1 were injected over the surface. The kinetic analysis between ROCK1 and TFEB was performed. d Schematic diagram of WT and truncated ROCK1 plasmids. e TFEB phosphorylation sites that promote its cytoplasmic retention.


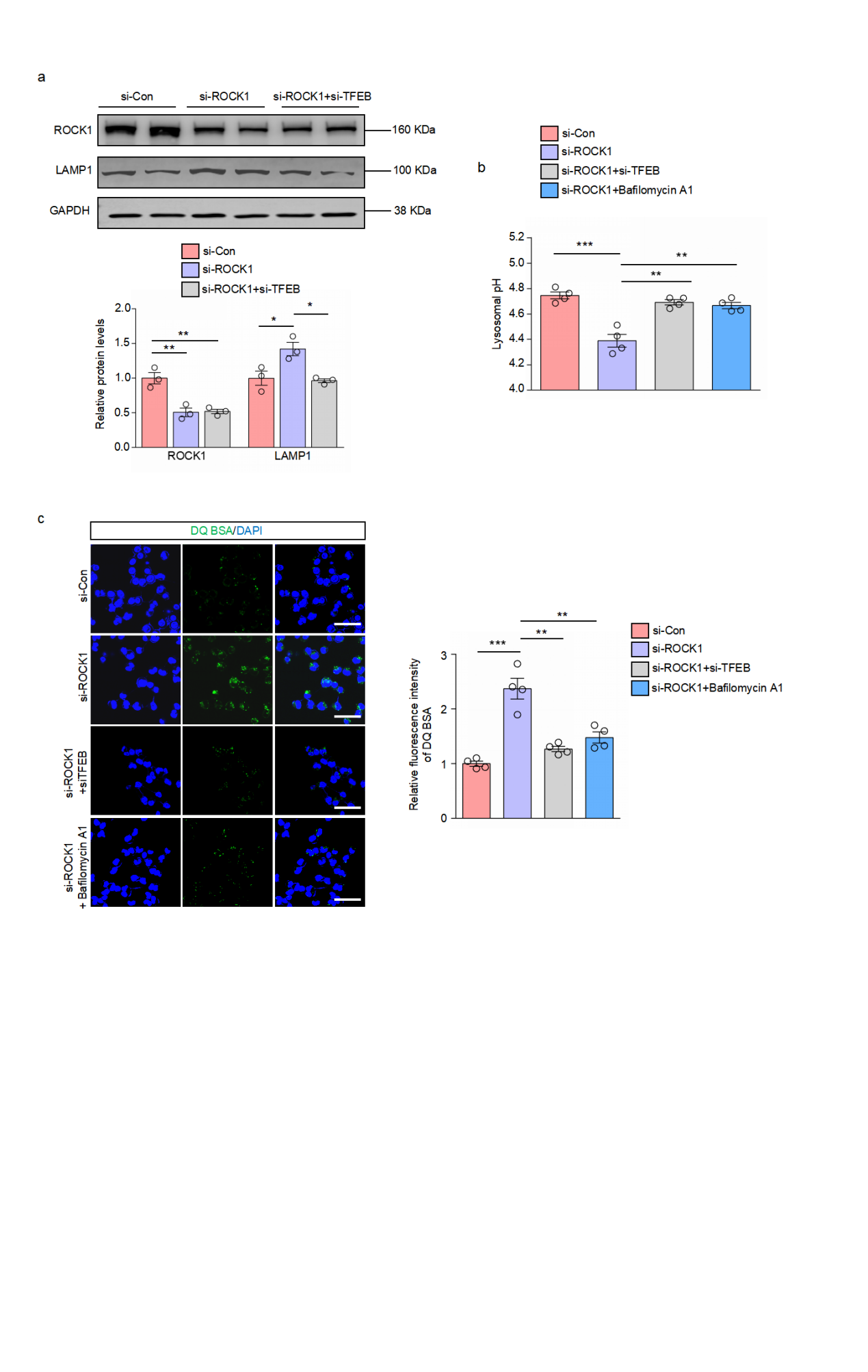


Figure S7 Downregulation of TFEB attenuates the regulatory effects of ROCK1 downregulation on lysosome. a HEK-293T cells were transfected with indicated siRNAs for 24 h, and the protein levels of ROCK1 and LAMP1 were detected by immunoblotting. *n =* 3. **P* < 0.05, ***P* < 0.01 vs indicated group. b HEK-293T cells were pre-incubated with Bafilomycin A1 (100 nM 1 h), and then transfected with si-Con/si-ROCK1 for 24 h. Lysosomal pH was determined by LysoSensor. *n =* 4. ***P* < 0.01, ****P* < 0.001 vs indicated group. c HEK-293T cells were pre-incubated with Bafilomycin A1 (100 nM 1 h), and then transfected with si-Con/si-ROCK1 for 24 h. Degradation of lysosome-loaded DQ BSA was analyzed. *n =* 4. ***P* < 0.01, ****P* < 0.001 vs indicated group. Scale bar, 50 μm.


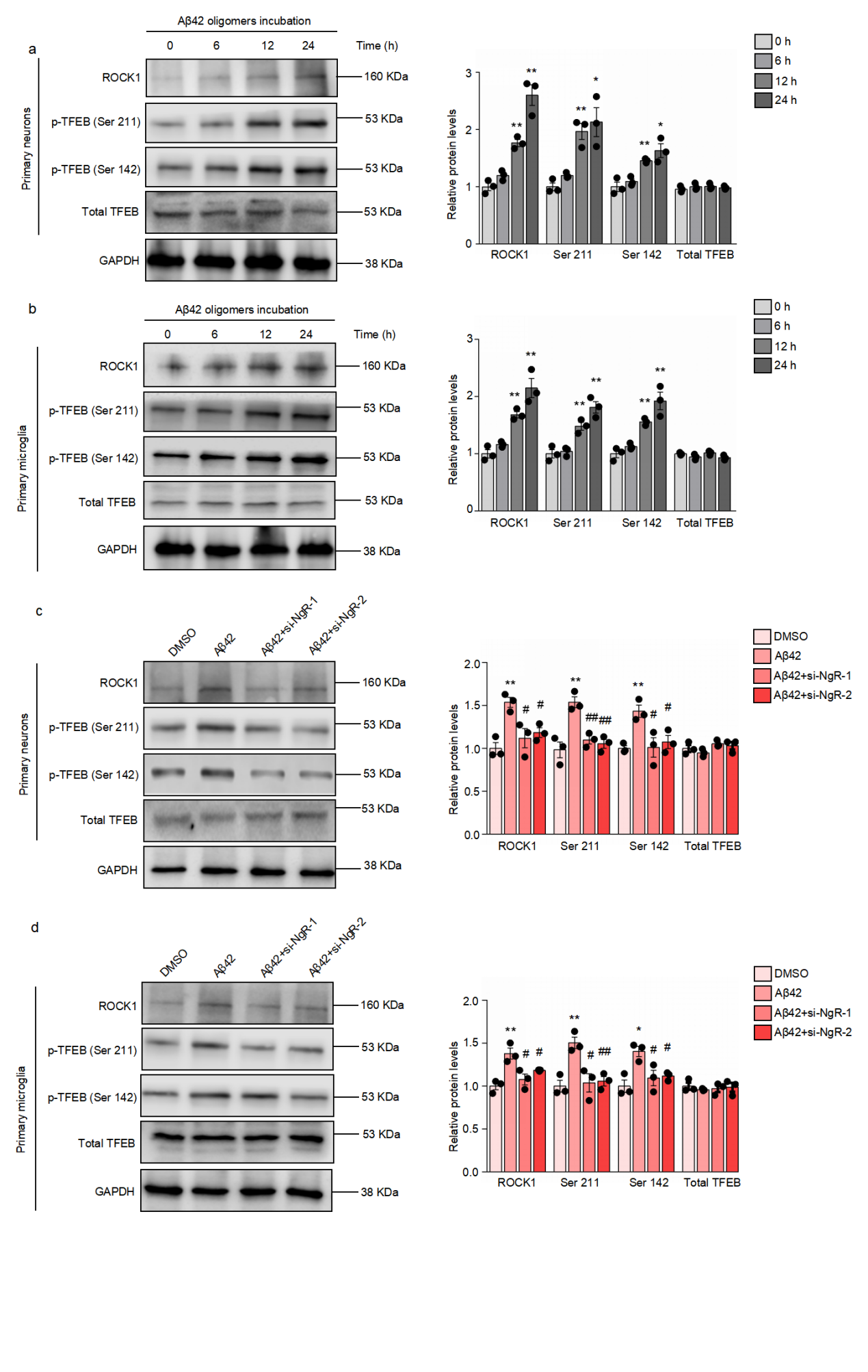


Figure S8 Aβ increases ROCK1 and phosphorylated TFEB levels to impair lysosomal function. a-b Primary mouse neurons (a) and microglia (b) were incubated with 5 μM Aβ_42_ oligomers for different time, and the protein levels of ROCK1 and phosphorylated TFEB were detected by immunoblotting. *n =* 3. **P* < 0.05, ***P* < 0.01 vs indicated 0 h group. c-d Primary mouse neurons (a) and microglia (b) were transfected with two independent NgR siRNAs for 24 h, followed by incubating with 5 μM Aβ_42_ oligomers for 12 h. ROCK1 and phosphorylated TFEB protein levels were detected by immunoblotting. *n =* 3. **P* < 0.05, ***P* < 0.01 vs indicated DMSO group, ^#^*P* < 0.05, ^##^*P* < 0.01 vs indicated Aβ_42_ group. ROCK1 and total TFEB levels were normalized by GAPDH and phosphorylated TFEB levels were normalized by total TFEB.


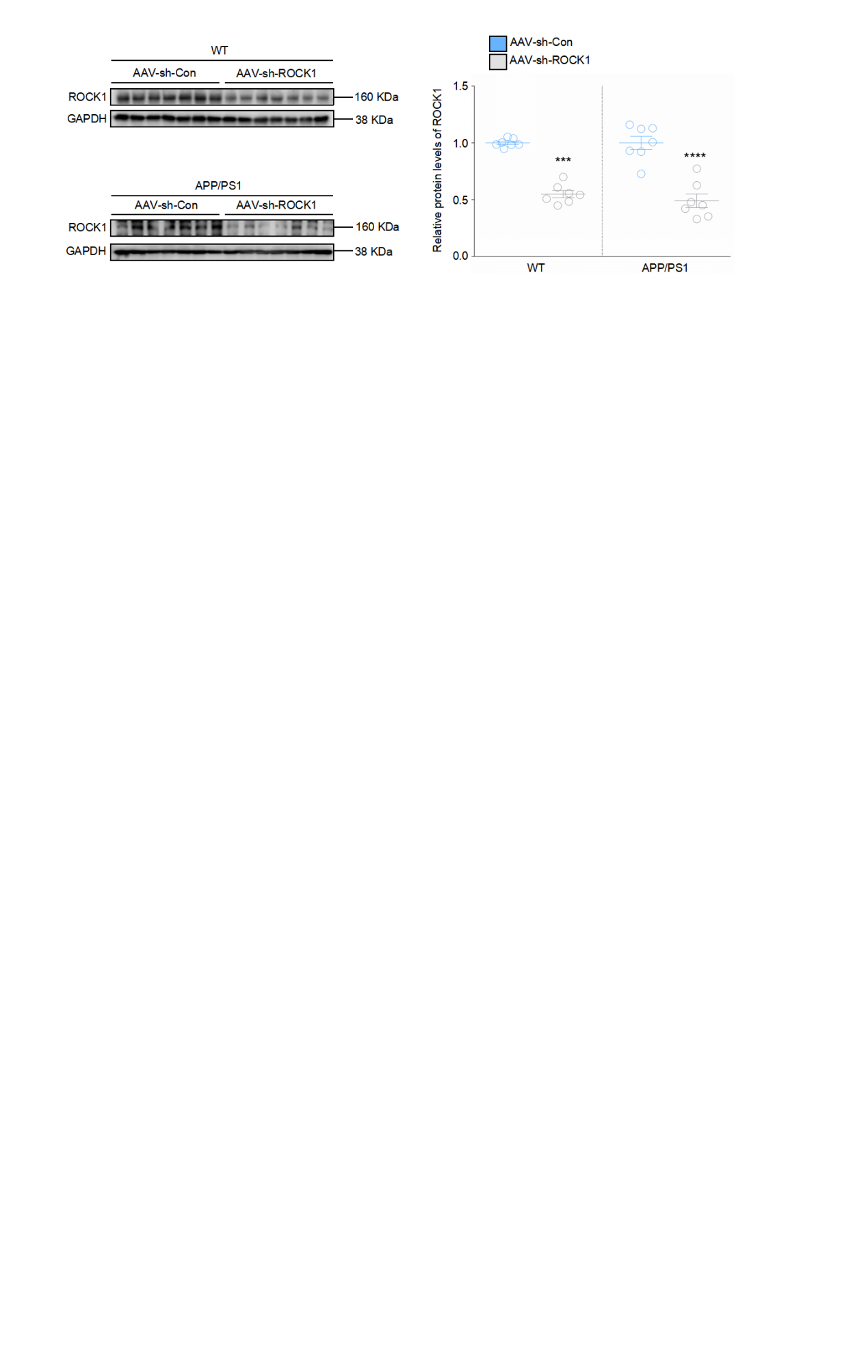


Figure S9 Knockdown efficiency of ROCK1 shRNA in the hippocampus of WT and APP/PS1 mice. Immunoblotting was carried out to detect the protein levels of ROCK1 in the hippocampus of WT and APP/PS1 mice microinjected with AAV-sh-Con/AAV-sh-ROCK1. *n =* 7. ****P* < 0.001, *****P* < 0.0001 vs AAV-sh-Con group using Student’s *t*-test.


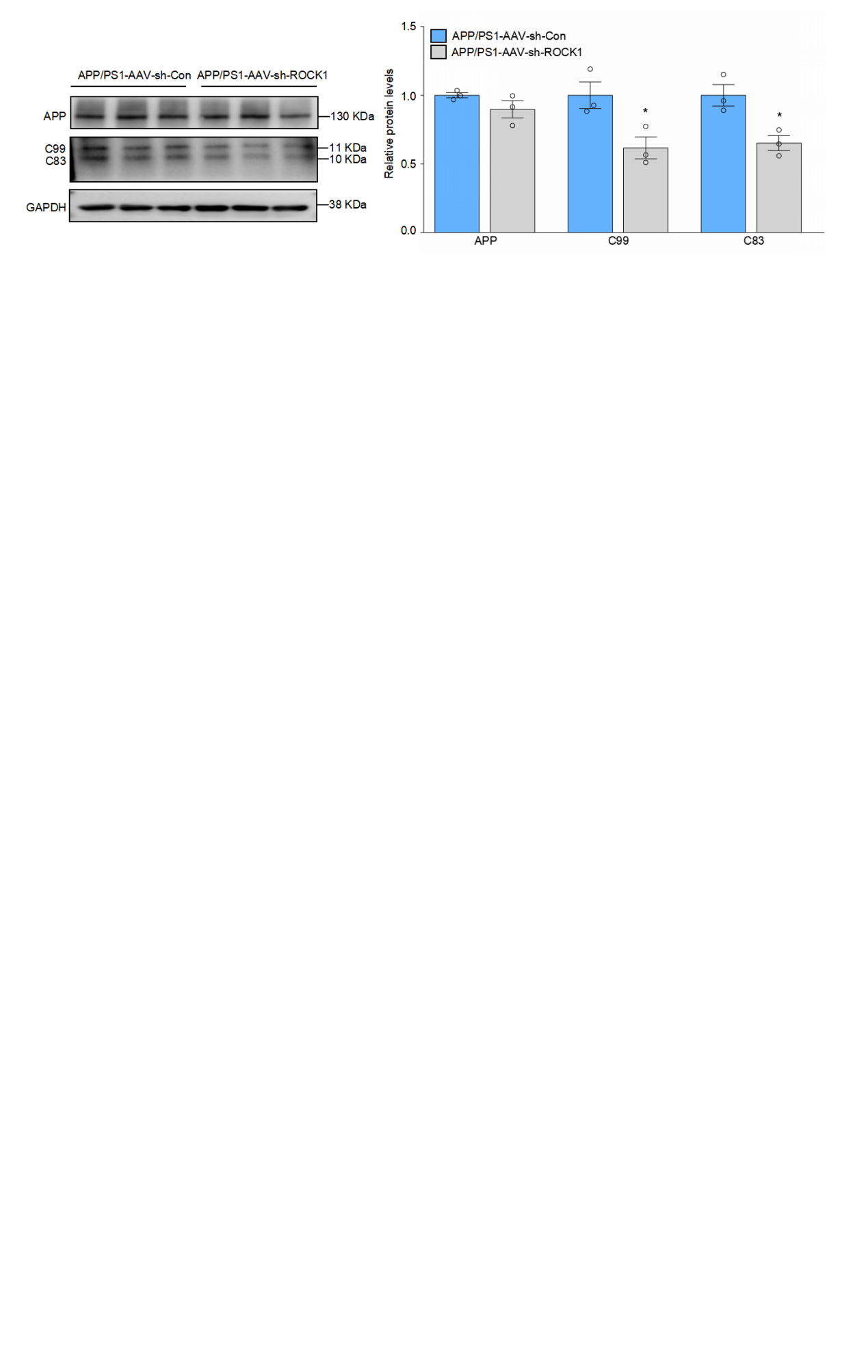


Figure S10 ROCK1 downregulation inhibits amyloidogenic processing of APP in the brain of APP/PS1 mice. Immunoblotting was carried out to detect the protein levels of APP, C99 and C83 in the hippocampus of APP/PS1 mice microinjected with AAV-sh-Con/AAV-sh-ROCK1. *n =* 3. **P* < 0.05 vs APP/PS1-AAV-sh-Con group using Student’s *t*-test.
